# mTOR Directs Breast Morphogenesis through the PKC-alpha-Rac1 Signaling Axis

**DOI:** 10.1371/journal.pgen.1005291

**Published:** 2015-07-01

**Authors:** Meghan M. Morrison, Christian D. Young, Shan Wang, Tammy Sobolik, Violeta M. Sanchez, Donna J. Hicks, Rebecca S. Cook, Dana M. Brantley-Sieders

**Affiliations:** 1 Department of Cancer Biology, Vanderbilt University School of Medicine, Nashville, Tennessee, United States of America; 2 Department of Medicine, Vanderbilt University School of Medicine, Nashville, Tennessee, United States of America; 3 Vanderbilt-Ingram Cancer Center, Vanderbilt University School of Medicine, Nashville, Tennessee, United States of America; National Cancer Institute, UNITED STATES

## Abstract

Akt phosphorylation is a major driver of cell survival, motility, and proliferation in development and disease, causing increased interest in upstream regulators of Akt like mTOR complex 2 (mTORC2). We used genetic disruption of *Rictor* to impair mTORC2 activity in mouse mammary epithelia, which decreased Akt phosphorylation, ductal length, secondary branching, cell motility, and cell survival. These effects were recapitulated with a pharmacological dual inhibitor of mTORC1/mTORC2, but not upon genetic disruption of mTORC1 function via *Raptor* deletion. Surprisingly, Akt re-activation was not sufficient to rescue cell survival or invasion, and modestly increased branching of mTORC2-impaired mammary epithelial cells (MECs) in culture and *in vivo*. However, another mTORC2 substrate, protein kinase C (PKC)-alpha, fully rescued mTORC2-impaired MEC branching, invasion, and survival, as well as branching morphogenesis *in vivo*. PKC-alpha-mediated signaling through the small GTPase Rac1 was necessary for mTORC2-dependent mammary epithelial development during puberty, revealing a novel role for Rictor/mTORC2 in MEC survival and motility during branching morphogenesis through a PKC-alpha/Rac1-dependent mechanism.

## Author Summary

The protein kinase mTOR is frequently activated in breast cancers, where it enhances cancer cell growth, survival, and metastastic spread to distant organs. Thus, mTOR is an attractive, clinically relevant molecular target for drugs designed to treat metastatic breast cancers. However, mTOR exists in two distinct complexes, mTORC1 and mTORC2, and the relative roles of each complex have not been elucidated. Moreover, as pathways that regulate normal tissue growth and development are often highjacked to promote cancer, understanding mTOR function in normal mammary epithelial development will likely provide insight into its role in tumor progression. In this study, we assessed the role of mTORC1 and mTORC2 complexes in normal mammary epithelial cell branching, survival, and invasion. Interestingly, while mTORC1 was not required for branching, survival and invasion of mammary epithelial cells, mTORC2 was necessary for these processes in both mouse and human models. Furthermore, we found that mTORC2 exerts its effects primarily through downstream activation of a PKC-alpha-Rac1 signaling axis rather than the more well-studied Akt signaling pathway. Our studies identify a novel role for the mTORC2 complex in mammary morphogenesis, including cell survival and motility, which are relevant to breast cancer progression.

## Introduction

Post-natal mammary epithelial morphogenesis is a complex process during which an extensively branched ductal network develops from a rudimentary epithelial bud [1]. Branching morphogenesis is most active during puberty and is regulated by endocrine hormones and local paracrine interactions with mesenchymal stroma [2]. In response to hormonal and growth factor cues, mammary epithelial cells (MECs) within the terminal end buds (TEBs), the club-shapes structures at the distal epithelial tips [1, 2], proliferate and collectively invade surrounding stroma. Differentiation of epithelial progenitors in the TEB populates the ducts with mature luminal MECs, and apoptosis canalizes the lumen. TEB bifurcation results from mechanical restraints at the TEB midline, forming new primary ducts. Side-branches sprout laterally from the trailing ducts as proliferative out-pouchings. Primary and side branching reiterates, filling the entire mammary fat pad [1, 2]. The dynamic processes that occur during puberty in the mammary epithelium are carefully coordinated by many molecular signaling pathways.

The intracellular serine/threonine kinase mammalian target of rapamycin (mTOR) regulates cellular metabolism, protein and lipid synthesis, cell survival, and cytoskeletal organization, processes that are required for proper mammary morphogenesis. mTOR regulates these processes through phosphorylation of its target substrates, including translation initiation factor 4E (eIF4E)-binding protein 1 (4E-BP1), p70S6 kinase (S6K), Akt, SGK1, and protein kinase C-alpha (PKC-alpha) [3]. A complex of associated protein co-factors regulates mTOR substrate specificity. As such, mTOR functions in two distinct complexes, each defined by the specific co-factors in complex with mTOR kinase and by their relative sensitivity to rapamycin. The rapamycin-sensitive mTOR complex (mTORC)-1 requires the co-factor regulatory-associated protein of mammalian target of rapamycin (Raptor), whereas mTORC2 requires the co-factor rapamycin-insensitive companion of mammalian target of rapamycin (Rictor). Although mTORC2 is relatively insensitive to acute rapamycin treatment, more recent studies determined that prolonged rapamycin treatment can inhibit mTORC2 complex assembly [4–7]. The intracellular serine/threonine kinase Akt is phosphorylated at S473 directly by mTORC2 and is key effector for many of the biological effects initiated by mTORC2. Akt is also linked to activation of mTORC1 downstream of PI3-kinase, making Akt a point of intersection between mTORC1, mTORC2, and their associated effectors [3].

Though mTOR regulates MEC growth in cell lines [8, 9] and milk protein expression [8, 10–12], mTOR-mediated regulation of mammary ductal morphogenesis remains under-investigated. The signaling complexity of mTOR, its pleiotropic functions, and a lack of mTORC2-specific inhibitors present a challenge to dissecting the relative roles of mTORC1 and mTORC2 in mammary development. Given the importance of mTOR in breast cancer progression and treatment, an understanding of mTORC1 and mTORC2 in untransformed MECs is needed. We assessed the impact of tissue-specific *Rictor* and *Raptor* ablation on mammary morphogenesis. *Rictor* loss impaired mTORC2 activity, reduced ductal lengthening and secondary branching, and reduced MEC proliferation and survival *in vivo* and *ex vivo*. Surprisingly, genetic disruption of mTORC1 via *Raptor* ablation resulted in distinct and milder effects on the developing mammary ductal epithelium, revealing non-overlapping roles for mTORC1 and mTORC2 during mammary morphogenesis. Interestingly, we found that mTORC2 controls mammary morphogenesis through downstream effectors PKC-alpha and Rac1, but not Akt.

## Results

### Rictor/mTORC2 regulates ductal branching, lengthening, and cell survival in the mammary gland *in vivo*

**Fig 1:**
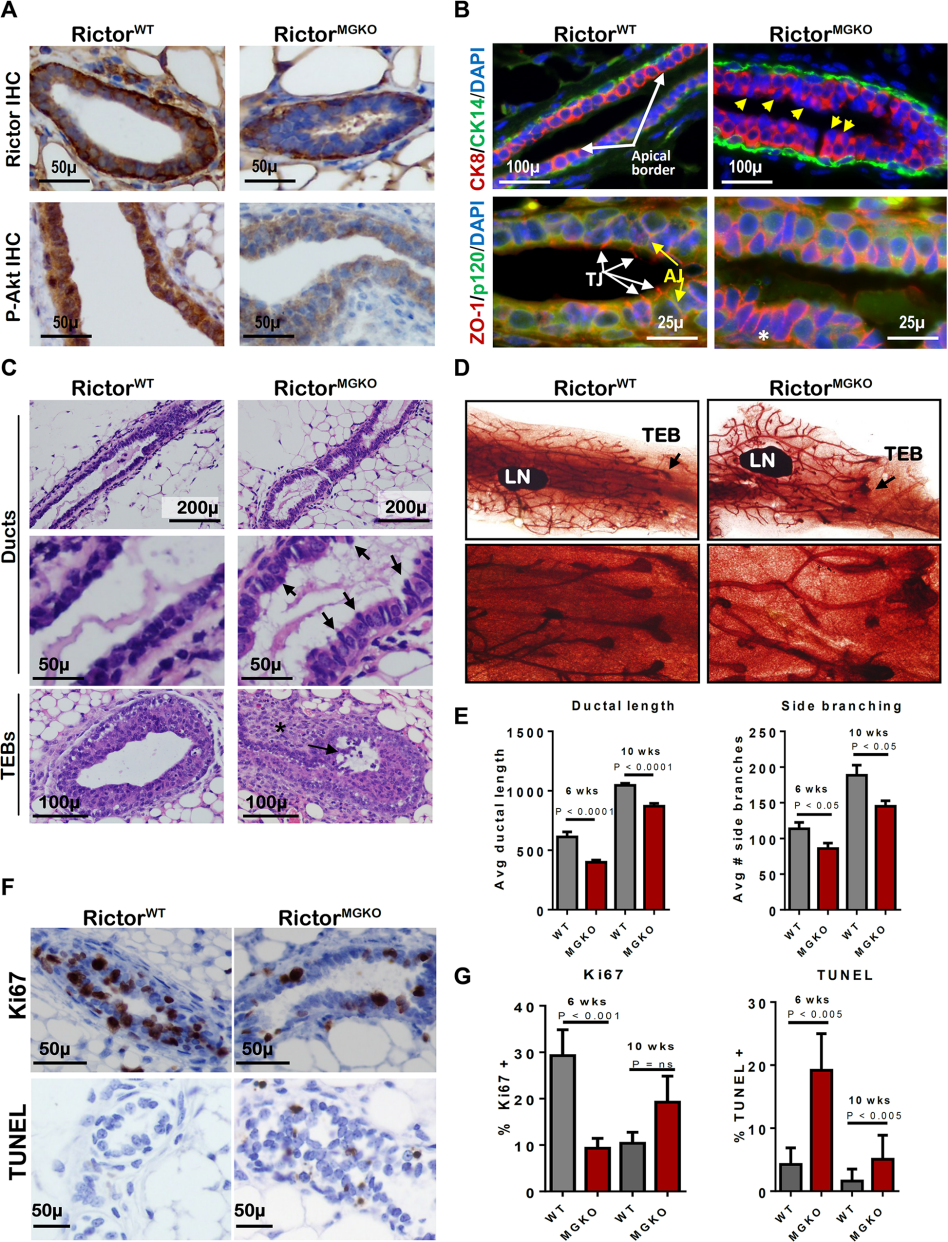
Loss of Rictor disrupts mammary branching morphogenesis in vivo. **A.** IHC for Rictor (upper panels) and P-Akt S473 (lower panels) in mammary gland sections from 6 week-old virgin mice. **B.** IF for CK8, CK14, ZO-1 and P120 in mammary gland sections. Yellow arrows indicate apically mis-localized nuclei in Rictor^MGKO^ tissue in upper right panel. White arrows indicate tight junctions (TJ) and yellow arrows indicate adherens junctions (AJ) in lower left panel. **C.** Hematoxylin and eosin (H&E)-stained mammary sections. Black arrows indicate irregular apical border in middle right panel. Black arrow indicates body cells sloughing in the TEB lumen and (*) indicates stromal thickening at the neck between maturing duct and TEB. **D.** Whole mount hematoxylin-stained mammary glands from 6-week virgin mice; lymph nodes (LN); terminal end buds (TEBs, arrows). **E.** Average ductal length (microns) beyond the mammary lymph node (left) and average number of side branches (right), ± S.D. **F.** IHC for Ki67 (upper panels) and TUNEL (lower panels) in mammary glands from 6-week old mice. Representative images are shown. **G.** Average percent Ki67+ nuclei (left) or TUNEL+ nuclei (right) per total MEC nuclei, ± S.D. N = 10 mice for each genotype/time point, Student’s T-test.

To assess the role of Rictor/mTORC2 during mammary morphogenesis in the context of the native mammary microenvironment, we bred *MMTV-Cre* mice [13] to *Rictor* ^*FL/FL*^ mice [14], allowing mammary-specific Cre recombinase to disrupt Rictor expression at floxed (FL) *Rictor* alleles. Immunohistochemistry (IHC) for Rictor revealed expression in luminal and myoepithelial MECs in *Rictor* ^*+/+*^ *MMTV-Cre* (*Rictor* ^*WT*^) mice (**Fig 1A–upper panel**). Rictor expression was not seen in *Rictor* ^*FL/FL*^ *MMTV-Cre* (*Rictor* ^*MGKO*^) luminal MECs, and was slightly reduced in the myoepithelium, consistent with luminal but not myoepithelial Cre expression in *MMTV-Cre* mice. Akt phosphorylation at S473, the mTORC2 phosphorylation site, was decreased in MECs of *Rictor* ^*MGKO*^ mice versus *Rictor* ^*WT*^, confirming decreased mTORC2 signaling upon Rictor ablation **(Fig 1A–lower panel)**. Immunofluorescent (IF) staining for cytokeratin (CK)-8 and CK14, molecular markers of luminal and myoepithelial MECs, respectively, confirmed that Rictor loss did not affect the relative spatial organization of luminal and myoepithelial MECs (**Fig 1B–upper panel**), but revealed the presence of apically mis-localized nuclei in *Rictor* ^*MGKO*^ MECs (yellow arrows), versus basally located nuclei and an organized, smooth apical border in *Rictor* ^*WT*^ samples (white arrows). IF for the tight junction (TJ) protein Zona Occludens-1 (ZO-1) revealed apical ZO-1 localization in *Rictor* ^*WT*^ samples. However, ZO-1 was aberrantly localized along baso-lateral membranes in *Rictor* ^*MGKO*^ MECs (**Fig 1B–lower panel**). In contrast, the baso-lateral localization of the adherens junction (AJ) protein p120 was relatively unaltered by Rictor loss. These results suggest that Rictor loss disrupts the proper apical distribution of ZO-1 in MECs. The apically mis-localized nuclei apparent in histological mammary sections from 6-week old *Rictor* ^*MGKO*^ female mice contributed to an irregular apical border (**Fig 1C**, **black arrows**). Additional structural alterations were seen in TEBs, including sloughing of body cells (the multi-layered TEB population comprised of mature and progenitor luminal MECs; **Fig 1C–lower panel, arrow**) within TEB lumens, and stromal thickening at the neck between maturing ducts and TEBs (Fig 1C–lower panel, *). Morphological alterations were seen throughout whole mounted, hematoxylin-stained *Rictor* ^*MGKO*^ mammary glands (**Figs 1D, arrows, and S1A**). Because mammary ducts lengthen distally at a predictable rate during puberty, we measured ductal length in mammary glands from 6 week- (mid-puberty) and 10 week-old (late puberty) mice. Ductal length was significantly reduced in *Rictor* ^*MGKO*^ mammary glands at both time points (**Fig 1E–left panel, and S1B Fig**). Primary (Y-shaped) and side (T-shaped) branches were counted in each mammary gland, revealing a significant reduction in T-shaped side branches at 6 and 10 weeks of age in *Rictor* ^*FL/FL*^ *MMTV-Cre* samples as compared to *Rictor* ^*WT*^ (**Fig 1E–right panel**).

IHC analysis of Ki67 in both ducts and TEBs was used as a relative measure of cellular proliferation in the mammary epithelium (**Figs 1F—upper panel, and S1C–upper panel**), revealing decreased Ki67+ nuclei in *Rictor* ^*MGKO*^ samples as compared to *Rictor* ^*WT*^ at 6 weeks of age but not at 10 weeks (**Fig 1G—left panel).** Cell death in ductal MECs or TEBs, measured using TUNEL analysis (**Figs 1F—lower panel, and S1C–lower panel**), demonstrated a remarkable increase in TUNEL+ MECs in *Rictor* ^*MGKO*^ samples at 6 and 10 weeks of age (**Fig 1G—right panel).** These results demonstrate that Rictor loss impairs mTORC2 activity, P-Akt, MEC growth, and MEC survival during mammary morphogenesis.

### Defects in MEC survival, branching, and motility are recapitulated by Rictor loss in an *ex vivo* model of mammary morphogenesis

**Fig 2:**
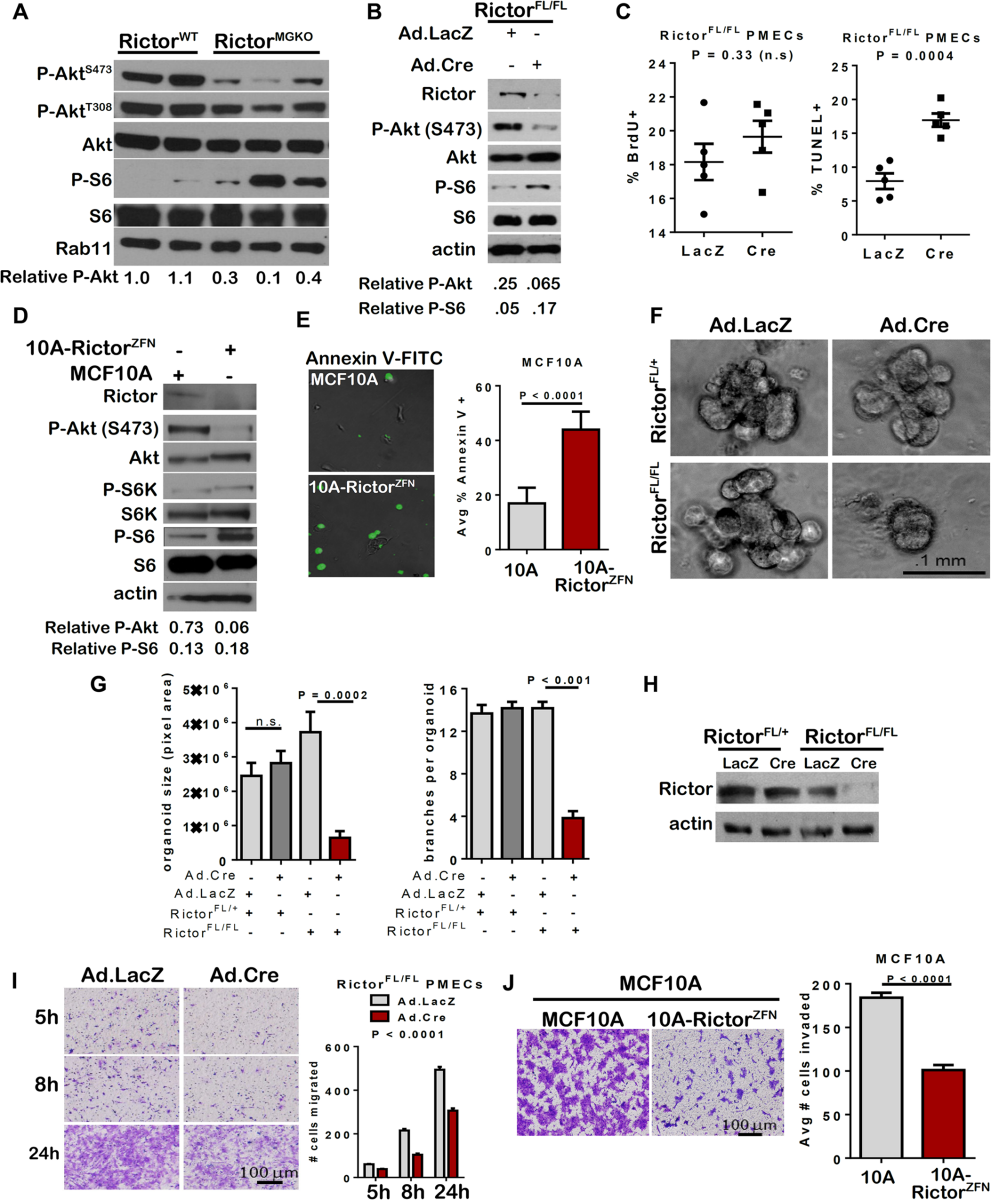
Impaired survival and morphogenesis of mammary epithelial structures upon loss of Rictor *ex vivo*. **A.** Western analysis of whole mammary lysates harvested from 10-week old female mice. **B-C.** *Rictor* ^*FL/FL*^ PMECs were infected with Ad.Cre or Ad.LacZ and cultured 7 days. **B.** Western analysis of PMEC lysates under serum starved conditions. Quantitation was performed using Image J software and numbers represent P-Akt or P-S6 bands normalized to total Akt or S6 levels. **C.** BrdU+ (left) and TUNEL+ (right) nuclei relative to total nuclei were quantified. N = 5 epithelial isolates, each analyzed in triplicate. Midline values indicate average, whiskers indicate S.D., Student’s T-test. D-E. MCF10A and MCF10A-Rictor^ZFN^ cells were analyzed. **D.** Western analysis of MCF10A lysates. **E.** Cells were labeled with Annexin V-FITC for 6 hours then photographed. Percent Annexin V+ cells versus total was quantified, average ± S.D. shown. N = 3 independent experiments, each analyzed in quadruplicate, representative images shown, Student’s T-test. **F-I.** *Rictor* ^*FL/FL*^ PMECs and organoids were infected with Ad.Cre or Ad.LacZ. **F.** Organoids photographed after 10 days in Matrigel culture. Representative images are shown. **G.** Average organoid size, measured in pixel area using Image J software, ± S.D. (left panel) and average number of branches/organoid ± S.D. (right panel) are shown. N = 6 independent organoid isolates, analyzed in triplicate, Student’s T-test. **H.** Western analysis of PMEC lysates. **I.** Transwell invasion of adenovirus-infected *Rictor* ^*FL/FL*^ PMECs in response to serum. Cells migrating to the opposite side of the transwell filter were visualized with crystal violet and counted. N = 6 PMEC isolates/condition, one-way ANOVA. **J.** Transwell invasion of MCF10A and MCF10A-Rictor^ZFN^ cells in response to serum is shown as average number of invading cells ± S.D. N = 3, independent experiments, each analyzed in triplicate, Student’s T-test.

Western analysis of whole mammary lysates harvested from 10-week old female mice confirmed decreased P-Akt S473 in *Rictor* ^*MGKO*^ mammary glands, and revealed increased phosphorylation of the mTORC1 effector ribosomal protein S6 ([15]; **Fig 2A**) confirming that Rictor loss decreases mTORC2 activity, but not mTORC1. To dissect more precisely how Rictor signaling affects mammary morphogenesis, we used primary mammary epithelial cells (PMECs) and primary mammary organoids (PMO’s) harvested from *Rictor* ^*FL/FL*^ mice. Adenoviral infection of *Rictor* ^*FL/FL*^ PMECs with Ad.Cre significantly reduced Rictor and P-Akt S473 levels relative to cells infected with control Ad.LacZ, and increased P-S6 levels (**Fig 2B**), similar to the impact of Rictor ablation *in vivo*. Consistent with structural alterations were seen in our *Rictor* ^*MGKO*^ model *in vivo* (e.g. sloughing of body cells in TEBs, irregular ductal tracts, multiple cell layers), confocal analysis of Rictor-deficient PMOs stained for E-cadherin revealed multiple cell layers in acinar structures and poor lumen formation relative to control PMOs infected with Ad.LacZ, which formed a well-defined lumen surrounded by a single layer of epithelial cells (**S1D Fig**). Rictor loss did not significantly impact PMEC proliferation, as measured by bromodeoxyuridine (BrdU) incorporation into genomic DNA (**Fig 2C–left panel**). However, the percentage of TUNEL+ PMEC nuclei was increased >2-fold following Ad.Cre infection (**Fig 2C–right panel**), consistent with increased cell death in Rictor-null MECs *in vivo*. Similar results were seen using MCF10A immortalized human MECs, in which *Rictor* gene targeting with *Rictor*-specific zinc finger nucleases (ZFNs) genetically impaired Rictor expression and decreased P-Akt S473 (**Fig 2D**), thus validating our findings in a human MEC model. Increased cell death was also seen in MCF10A-Rictor^ZFN^ cells as compared to parental MCF10A cells, as shown by AnnexinV-FITC binding (**Fig 2E**). Therefore, Rictor is necessary for mTORC2 signaling and cell survival in human and mouse MECs.

We cultured adenovirus transduced *Rictor* ^*FL/FL*^ mammary organoids in three-dimensional (3D) Matrigel to assess collective epithelial morphogenesis (**Fig 2F**). Mammary organoids accurately model epithelial autonomous molecular events of mammary morphogenesis in a stroma-free environment that preserves the native relationship between luminal and myoepithelial MECs and permits cell-cell and cell-matrix interactions in three dimensions [16]. GFP fluorescence in organoids infected with Ad.GFP or Ad.Cre-IRES-GFP confirmed efficient infection in basal and luminal cells of organoids **(S2A and S2B Fig)**. IF staining for pan-cytokeratin confirmed that organoids were epithelial-derived (**S2C Fig**). Ad.Cre infection of *Rictor* ^*FL/FL*^ PMECs substantially reduced organoid size and branching (**Fig 2F and 2G**) and reduced Rictor expression levels (**Fig 2H**). In contrast, Ad.Cre infection of *Rictor* ^*FL/+*^ PMECs only modestly reduced Rictor expression levels (**Fig 2H**) and did not significantly affect organoid size or the number of branches formed in *Rictor* ^*FL/+*^ organoids (**Fig 2F and 2G**). These data suggest that Rictor is necessary for multicellular morphogenesis of the mammary epithelium, faithfully recapitulating *ex vivo* the consequences of Rictor ablation that are seen *in vivo* and demonstrating the utility of this model to examine branching mammary gland morphogenesis.

Previous studies demonstrated that Rictor knock-down reduces migration of breast cancer cell lines [17–19]. We therefore assessed PMEC invasion and motility through Matrigel-coated transwell filters upon Rictor ablation *ex vivo*. Fewer *Rictor* ^*FL/FL*^ PMECs invaded through Matrigel when infected with Ad.Cre, as compared to *Rictor* ^*FL/FL*^ PMECs infected with control Ad.LacZ (**Fig 2I**). Similarly, invasion through Matrigel-coated transwells was profoundly reduced in MCF10A-Rictor^ZFN^ cells as compared to parental MCF10A cells (**Fig 2J**). Under these conditions, there were a similar number of viable cells remaining in the upper transwell chamber after 24 hours of culture of both MCF10A and MCF10A-Rictor^ZFN^ cells **(S2D Fig),** suggesting that cell death may not be the primary reason underlying the reduced ability of MCF10A-Rictor^ZFN^ cells to migrate/invade in these transwell assays, but rather that cell invasion, *per se*, is decreased in the absence of Rictor. Collectively, these data demonstrate that Rictor promotes MEC invasion and migration, two processes necessary for mammary ductal lengthening and branching.

### Akt activation, while necessary for normal branching morphogenesis, is not sufficient to rescue morphogenesis, survival, and motility defects in the absence of Rictor

**Fig 3:**
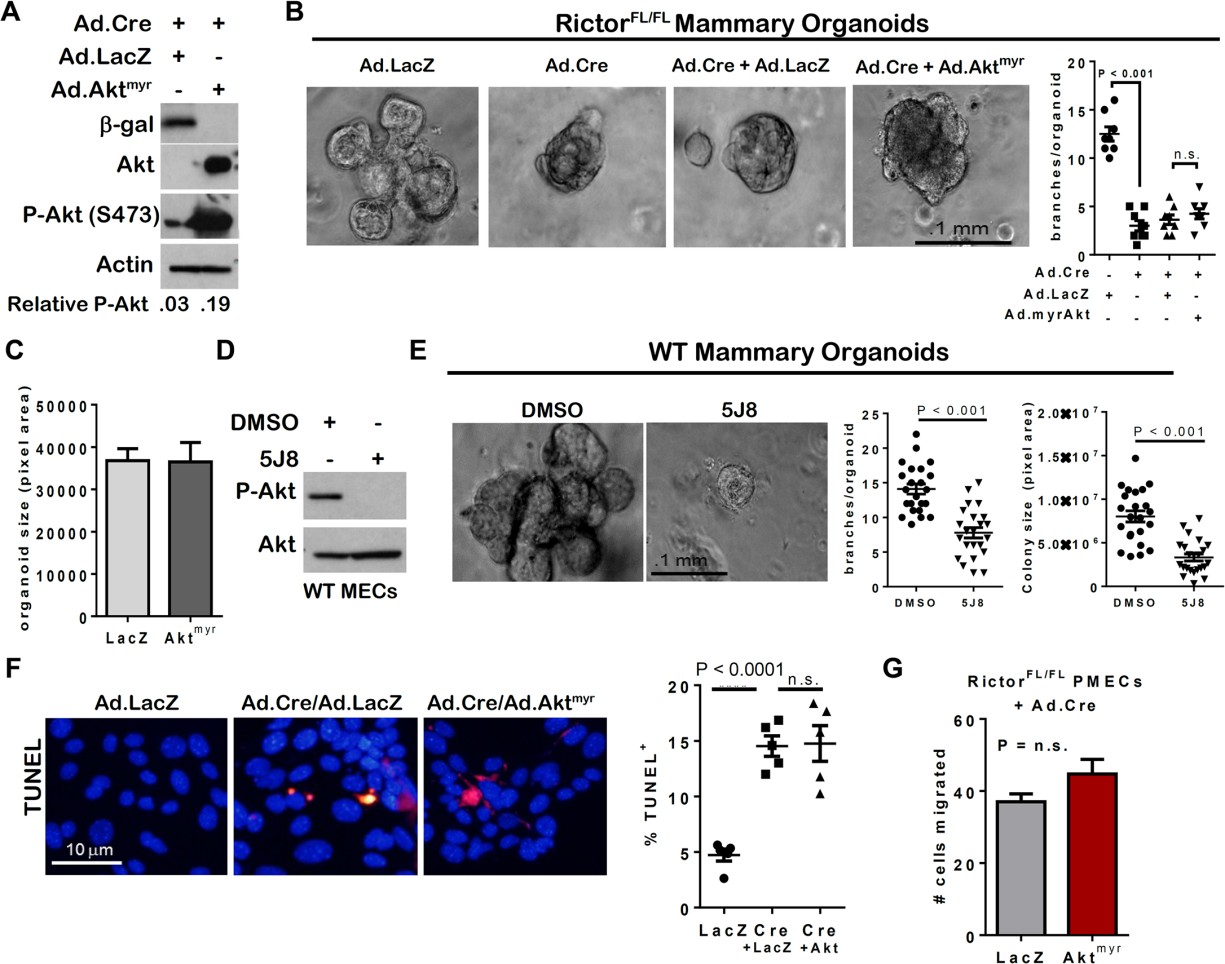
Akt activation is insufficient to rescue Rictor-deficient MEC survival, branch formation and invasion. **A-C.** PMECs and organoids from *Rictor* ^*FL/FL*^ mice were co-infected with Ad.Cre and either Ad.LacZ or Ad.Akt^myr^. **A.** Western analysis of PMEC lysates. **B.** Organoids photographed after 10 days in Matrigel culture. Average number of branches/organoid ± S.D. (right panel) are shown. N = 20 independent organoids from 5 independent mice. Midline values indicate average (data points = averages from individual experiments), whiskers indicate S.D., Student’s T-test. **C.** Colony size of organoids measured in pixel area. N = 20 independent organoids from 5 independent mice, average ± S.D., Student’s T-test. **D-E.** PMECs and organoids from *WT* mice treated 3–7 days with 5J8 Akt inhibitor or DMSO. **D**. Western analysis of PMECs after 3 days treatment with 5J8. **E.** Organoids photographed after 10 days in DMSO or 5J8. Average number of branches/organoid ± S.D. (left panel) are shown. Midline values indicate average (data points = averages from individual experiments), whiskers indicate S.D., Student’s T-test. Organoid size measured in pixel area shown in right panel Student’s T-test. N = 23 independent organoids from 6 independent mice. **F-G.** PMECs from *Rictor* ^*FL/FL*^ mice were co-infected with Ad.Cre and either Ad.LacZ or Ad.Akt^myr^. **F.** PMECs were assessed by TUNEL analysis. Representative images are shown. Average percent TUNEL+ nuclei (red) per total nuclei (DAPI, blue) was calculated. Individual cell isolates (N = 5, analyzed in duplicate) are represented by each data point. Midlines values are average percentage of TUNEL+ nuclei ±S.D., indicated by whiskers, Student’s T-test. **G.** PMECs migrating to the opposite side of Matrigel-coated transwell filters were visualized with crystal violet and counted. N = 6 independent cell isolates per condition, analyzed in duplicate, Student’s T-test.

Because Rictor loss reduced P-Akt S473, we tested the hypothesis that Akt phosphorylation by Rictor-regulated mTOR complex 2 is necessary for survival and morphogenesis of MECs. Adenoviral expression of myristoylated Akt1 (Ad.Akt^myr^) was used to express a membrane-localized (and thus, constitutively active) variant of Akt1. Indeed, expression of this Akt variant in mammary epithelium delays involution and the onset of apoptosis *in vivo* [20]. Additionally, we repeated experiments using an alternative adenoviral, constitutively active Akt construct, Ad.Akt^DD^. Ad.Akt^myr^ or Ad.Akt^DD^ restored P-Akt S473 in Ad.Cre-infected *Rictor* ^*FL/FL*^ PMECs (**Figs 3A and S3A**). Surprisingly, *Rictor* ^*FL/FL*^ organoids infected with Ad.Cre + Ad.Akt^myr^ or Ad.Akt^DD^ were morphologically similar to and harbored little to no statistically significant difference in the numbers of branches compared to those infected with Ad.Cre alone (**Figs 3B and S3B**). Further, size of Rictor-deficient organoids was not fully rescued by expression of Ad.Akt^myr^ or Ad.Akt^DD^ (**Figs 3C and S3C**). We found that blockade of Akt using the allosteric Akt inhibitor 5J8 blocked Akt phosphorylation at S473 (**Fig 3D**), reduced the number of branches per organoids, and reduced organoid size by nearly 50% (**Fig 3E**). These data suggest that while Akt is necessary for mammary branching and growth, restoring Akt function is not sufficient to completely rescue defects caused by loss of Rictor/mTORC2 function. Indeed, expression of Ad.Akt^myr^ did not reduce the number of Rictor-null PMECs undergoing cell death (**Fig 3F**), nor did it increase the number Rictor-null PMECs invading through Matrigel-coated transwells (**Fig 3G**). Taken together, these observations suggest Rictor is necessary for Akt phosphorylation in MECs, but that Akt is not the primary effector of mTORC2 that regulates MEC survival, invasion, and side branching. Thus, while Akt is necessary for proper mammary epithelial morphogenesis, it is not sufficient to compensate for loss of Rictor/mTORC2 function.

### Protein kinase C (PKC)-alpha activates the small GTPase Rac1 downstream of Rictor

**Fig 4:**
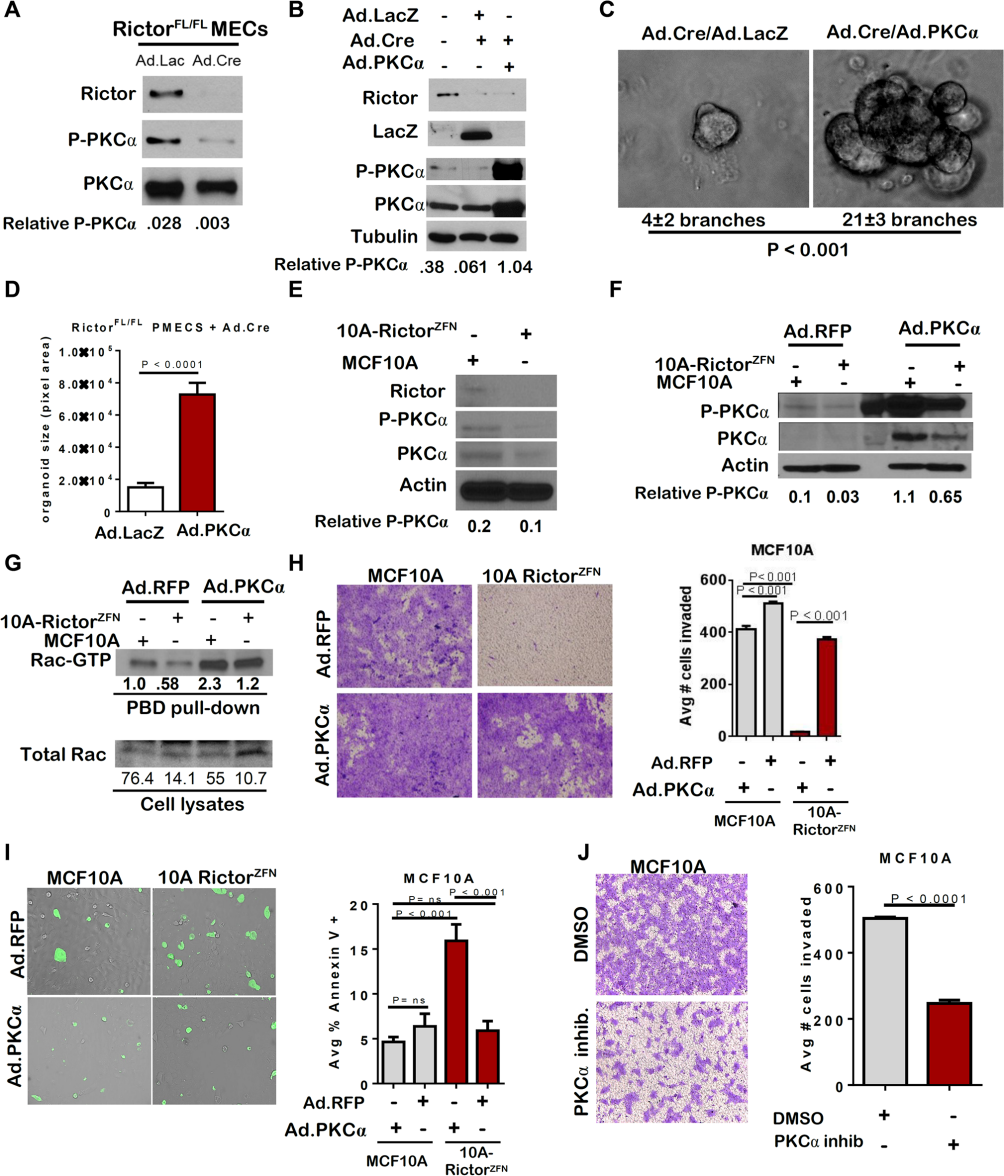
Loss of PKC-alpha-mediated Rac activation in the absence of Rictor. **A-D.** *Rictor* ^*FL/FL*^ PMECs and organoids were infected with the indicated adenoviruses. **A-B.** Western analysis of PMEC lysates. Quantitation was performed using Image J software and numbers represent P-PKC-alpha bands normalized to total PKC-alpha levels. **C**. Organoids photographed after 10 days in Matrigel culture. Average number of branches/organoid ±S.D. indicated below panels. N = 3 independent organoid isolates/condition analyzed in triplicate, Student’s T-test. **D.** Average colony size of organoids measured in pixel area ± S.D. N = 3 independent organoid isolates/condition, analyzed in triplicate, Student’s T-test**. E.** Western analysis of MCF10A parental and Rictor^ZFN^ lysates. Quantitation was performed using Image J software and numbers represent P-PKC-alpha band normalized to actin for each sample. **F-I.** MCF10A parental and Rictor^ZFN^ cells were infected with Ad.RFP or Ad.PKC-alpha. **F.** Western analysis of MCF10A parental and Rictor^ZFN^ lysates, 48 hours post-infection. Quantitation was performed using Image J software and numbers represent P-PKC-alpha band normalized to actin for each sample. **G.** GST-Pak-PBD effector pull-downs followed by western analysis for Rac performed 48 hours post-infection. Quantitation was performed using Image J software. Numbers in upper panel represent Rac-GTP levels in MCF10A-Rictor^ZFN^ compared to MCF10A parental control. Lower numbers represent Rac-GTP levels normalized to total Rac for each sample. **H.** Cells were assessed for invasion through Matrigel-coated transwell filters. Cells were stained with crystal violet after 24 hours, and then imaged. Number of cells invading was quantitated in Image J. Values shown represent the average ± S.D., Student’s T-test. N = 2 independent experiments, analyzed in triplicate. **I.** Cells were assessed for Annexin V-FITC. Number of Annexin V-FITC+ per total number of cells was quantitated in Image J, Student’s T-test. N = 2 independent experiments, analyzed in triplicate. **J.** MCF10A parental cells were assessed for invasion through Matrigel-coated transwell filters in the presence of PKC-alpha inhibitor GO6976. Cells were stained with crystal violet after 24 hours, and then imaged. Number of cells invading was quantitated in Image J. Values shown represent the average ± S.D., Student’s T-test. N = 2 independent experiments, analyzed in quadruplicate.

Previous studies showed that mTORC2 phosphorylates PKC-alpha [21] Consistent with these findings, Rictor loss reduced P-PKC-alpha in PMECs, as well as total PKC-alpha (**Fig 4A**). We also observed decreased P-PKC-alpha by IF in mammary gland sections from 6 week old *Rictor* ^*MGKO*^ mice, as compared to *Rictor* ^*WT*^ controls (**S4A Fig**). Adenoviral PKCα expression rescued P-PKC-alpha in Rictor-null PMECs (**Fig 4B**), rescued branching morphogenesis in Rictor-null organoids (**Fig 4C**) and increased Rictor-null organoid size (**Fig 4D**). Similar to what was seen in mouse PMECs, P-PKC-alpha and total PKC-alpha were diminished in MCF10A-Rictor^ZFN^ cells relative to parental MCF10A (**Fig 4E**). Restoration of PKC-alpha by adenoviral transduction increased P-PKC-alpha in both parental MCF10A and MCF10A-Rictor^ZFN^ cells (**Fig 4F**).

Rac1, a small GTPase involved in actin cytoskeletal dynamics, is necessary for migration of many breast cancer cell lines, regulates apical polarity in MECs, and is a downstream effector of mTORC2 signaling. Importantly, Rac1 is also a known effector of PKC-alpha in MECs [22–24], but the linear relationship between Rictor, PKC-apha, and Rac1 in MECs is currently unknown. We examined Rac1 activation in MCF10A cells using agarose beads conjugated to recombinant p21-activated kinase binding domain (PBD), which specifically binds to active GTP-bound Rac. Western analysis to detect Rac1 in PBD pull-downs revealed decreased Rac-GTP in MCF10A-Rictor^ZFN^ cells as compared to parental MCF10A (**Fig 4G**). However, Ad.PKC-alpha increased Rac-GTP in Rictor-null cells, confirming that PKC-alpha activates Rac downstream of Rictor. Additionally, Ad.PKC-alpha increased invasion of MCF10A-Rictor^ZFN^ cells through Matrigel-coated transwells (**Fig 4H**), and significantly reduced apoptosis in MCF10A-Rictor^ZFN^, as measured by Annexin V-FITC staining (**Fig 4I**). A pharmacological PKC-alpha inhibitor profoundly decreased invasion of parental MCF10A cells through Matrigel-coated transwells **(Fig 4J)**, providing validation that PKC-alpha is necessary for MEC motility. These data suggest Rictor-mediated PKC-alpha signaling in MECs controls Rac1 activation, branching morphogenesis, cell survival and motility.

### Restoring Rac activity rescues branching morphogenesis, survival, and motility induced by loss of Rictor

**Fig 5:**
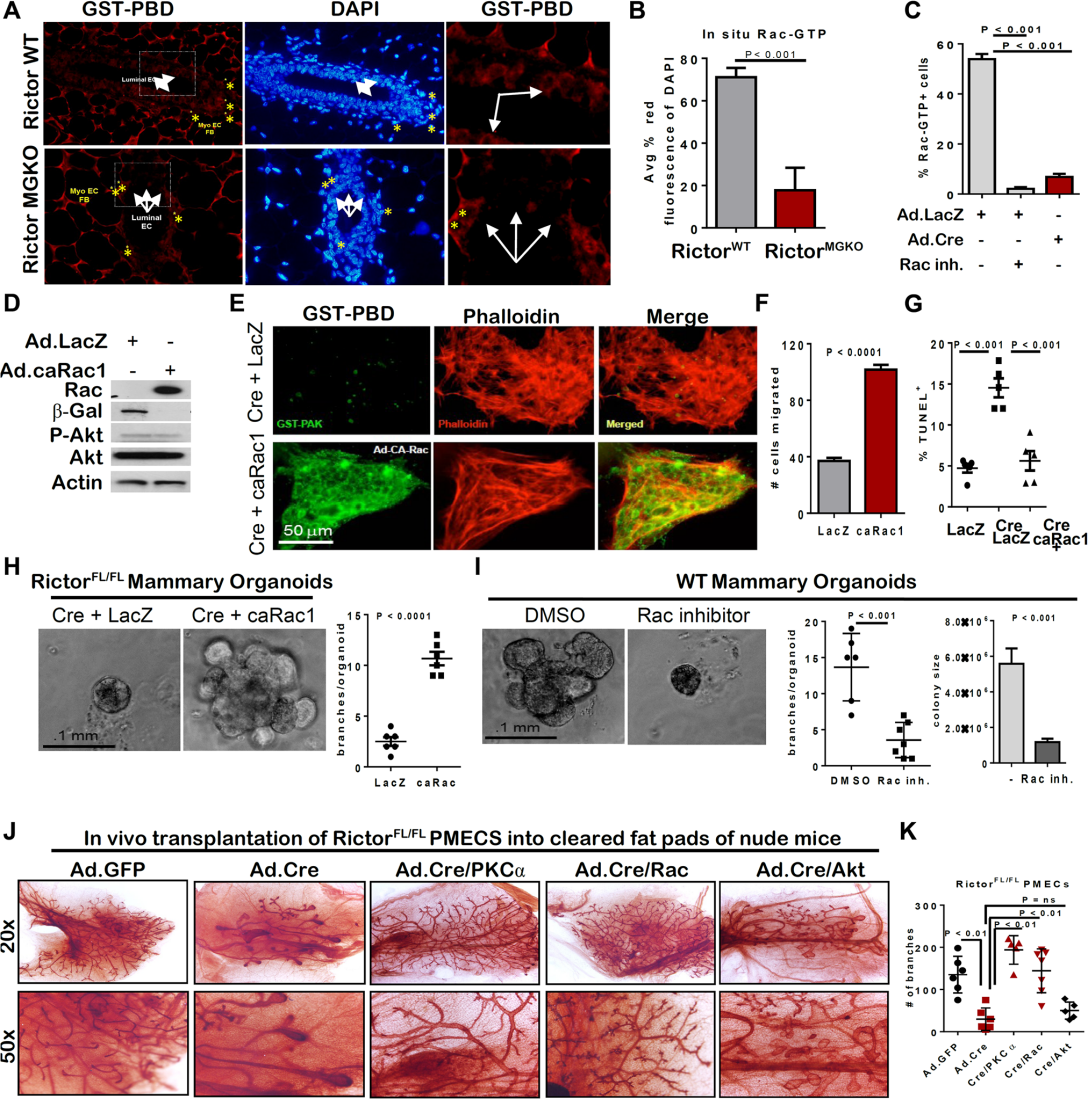
Rictor-mediated Rac activity is necessary and sufficient for mammary branching morphogenesis *ex vivo.* **A.** *In situ* detection of GTP-bound Rac via IF detection of GST-PBD (red; nuclei stained with DAPI, blue) on mammary gland sections from 6-week old mice. N = 6 independent fields analyzed in sections from 3 independent mammary gland sections/genotype. **B**. Quantitation of average percent red fluorescence (Rac-GTP) relative to blue (DAPI) on mammary gland sections from 6 week old virgin *Rictor* ^*WT*^ and *Rictor* ^*MGKO*^ mice, ± S.D., Student’s T-test. **C.** Cells were probed with GST-PBD (red) and counterstained with DAPI (blue). GST alone (not conjugated to PBD) was used as a negative control. GST-PBD+ fraction of total PMECs was counted and average ± S.D. shown. N = 15 fields/condition, Student’s T-test. **D-I** *Rictor* ^*FL/FL*^ PMECs infected with Ad.Cre or Ad.LacZ (±Ad.caRac1) and analyzed. **D.** Western analysis of PMEC lysates. **E.** Cells were probed with GST-PBD (green) and counterstained with phalloidin (red). **F.** Transwell invasion assays were performed. Invading cells were visualized with crystal violet and counted ±S.D. N = 6 independent isolates, analyzed in duplicate, Student’s T-test. **G.** PMECs were assessed by TUNEL analysis. N = 3 independent cell isolates, analyzed in duplicate. Representative images shown. Average percent TUNEL+ nuclei per total epithelial nuclei quantified. Midline values indicate average, whiskers indicate S.D., Student’s T-test. **H.** *Rictor* ^*FL/FL*^ organoids were infected Ad.Cre or Ad.LacZ (±Ad.caRac1) prior to embedding, and were photographed after 10 days in Matrigel culture. Right panel shows average number of branches per organoid. Each data point is the average branches per colony from individual isolates, analyzed in triplicate. Midlines are the average of all isolates, whiskers indicate S.D., Student’s T-test. N = 6. **I.** *WT* organoids were cultured ±Rac inhibitor for 10 days. Number of branches per organoid quantified. Each data point is the average branches per colony from individual isolates, analyzed in triplicate. Midline values indicate average, whiskers indicate S.D., Student’s T-test. Student’s T-test. Right panel shows average organoid size ±S.D. **J-K.** PMECs from *Rictor* ^*FL/FL*^ mice were tranduced with control Ad.GFP versus Ad.Cre in the presence or absence of Ad.PKC-alpha, Ad.caRac, or Ad.Akt^myr^. PMECs were transplanted into the cleared inguinal fat pads of 4 week old recipient female mice. Mammary glands were harvested six weeks post-transplantation and epithelial architecture and branching morphogenesis in whole-mount preparations was assessed. **J.** Whole-mount preparations from indicated transplanted glands. **K.** Quantitation of average number of branches ± S.D., Student’s T-test. Representative images shown from N = 2 independent experiments.

To confirm the role of Rictor in Rac1 activation *in vivo*, we examined mammary epithelium *in situ* for GTP-bound Rac1 using a glutathione-S-transferase (GST)-PBD fusion protein as a probe for Rac-GTP. IF detection of GST-PBD binding was decreased in *Rictor* ^*MGKO*^ mammary glands compared to *Rictor* ^*WT*^ **(Figs 5A, 5B, and S4B).** Importantly, IF detection of GST-PBD binding in WT PMECs was abolished by a pharmacological Rac1 inhibitor (**Figs 5C and S4C**), confirming the specificity of the assay for detection of Rac1-GTP. In contrast to the abundant Rac1-GTP detected in WT PMECs, *Rictor* ^*FL/FL*^ PMECs infected with Ad.Cre displayed a 10-fold decrease in GST-PBD binding to Rac-GTP relative to Ad.LacZ infected controls (**Figs 5C and S4C**). Phalloidin staining revealed cortical actin overlapping with GST-PBD binding in Ad.LacZ-infected *Rictor* ^*FL/FL*^ PMECs (**S4D Fig**). However, Ad.Cre-infected *Rictor* ^*FL/FL*^ PMECs showed increased formation of actin stress fibers, bearing no overlap with GST-PBD. Constitutively active Rac1 (Ad.caRac1) expression (**Fig 5D**) restored GST-PBD binding in Rictor-null PMECs (**Fig 5E**), suggesting that Rictor is necessary for Rac1-GTP in PMECs.

Ad.caRac1 was used to determine if restoration of Rac1-GTP could rescue invasion in Rictor-null MECs. Ad.caRac1 increased invasion 2.5-fold over Ad.LacZ in Rictor-null PMECs (**Fig 5F**). P-Akt S473 was unaffected by caRac1 (**Fig 5D**), suggesting that while Akt and Rac1 are both effectors of Rictor-dependent signaling, they exist in two separable pathways in MECs. Despite having no impact on P-Akt, Ad.caRac1 decreased cell death in Rictor-null PMECs (**Figs 5G and S4E**), suggesting that Rictor-dependent Rac1-GTP is necessary for PMEC survival. Ad.caRac1 also rescued branching morphogenesis of Rictor-deficient organoids (**Fig 5H**). Conversely, Rac inhibition using a pharmacologic Rac1 inhibitor decreased organoid size and branching in *WT* organoids (**Fig 5I**), confirming that Rac1 is necessary for mammary epithelial branching morphogenesis and appears to function downstream of mTORC2. Thus, Rictor is required for Rac1-GTP signaling, and restoration of Rac1 activity rescued branching morphogenesis and survival of Rictor-deficient PMECs. To determine if Rictor/mTORC2-mediated branching morphogenesis and ductal outgrowth are dependent on PKC-alpha/Rac versus Akt in the context of the native mammary gland environment *in vivo*, we transduced PMEC from *Rictor* ^*FL/FL*^ mice with control Ad.GFP versus Ad.Cre in the presence or absence of Ad.PKC-alpha, Ad.caRac, or Ad.Akt^myr^ and transplanted them into the cleared inguinal mammary fat pads of 4 week old recipient female mice. We harvested mammary glands from these animals 6 weeks post-transplantation and assessed epithelial architecture and branching morphogenesis in whole-mount preparations. Consistent with data from *MMTV-Cre/Rictor* ^*FL/FL*^ mice, transplanted Rictor-deficient MEC produced structures characterized by shortened ductal outgrowths with fewer branches relative to GFP controls **(Fig 5J and 5K).** Restored PKC-alpha or Rac activity **(Fig 5J and 5K)** rescued these defects and produced epithelial outgrowth that resembled endogenous epithelium in contralateral controls (**S4F Fig**). Consistent with our *ex vivo* organoid culture analyses, restored Akt activity was unable to fully rescue defects produced by loss of Rictor **(Fig 5J and 5K)**. These data suggest that Rictor/mTORC2-dependent mammary epithelial morphogenesis relies primarily upon downstream activation of PKC-alpha and Rac-GTPase.

### Rapamycin-mediated inhibition of mTORC1 and mTORC2 mimics loss of Rictor/mTORC2

**Fig 6:**
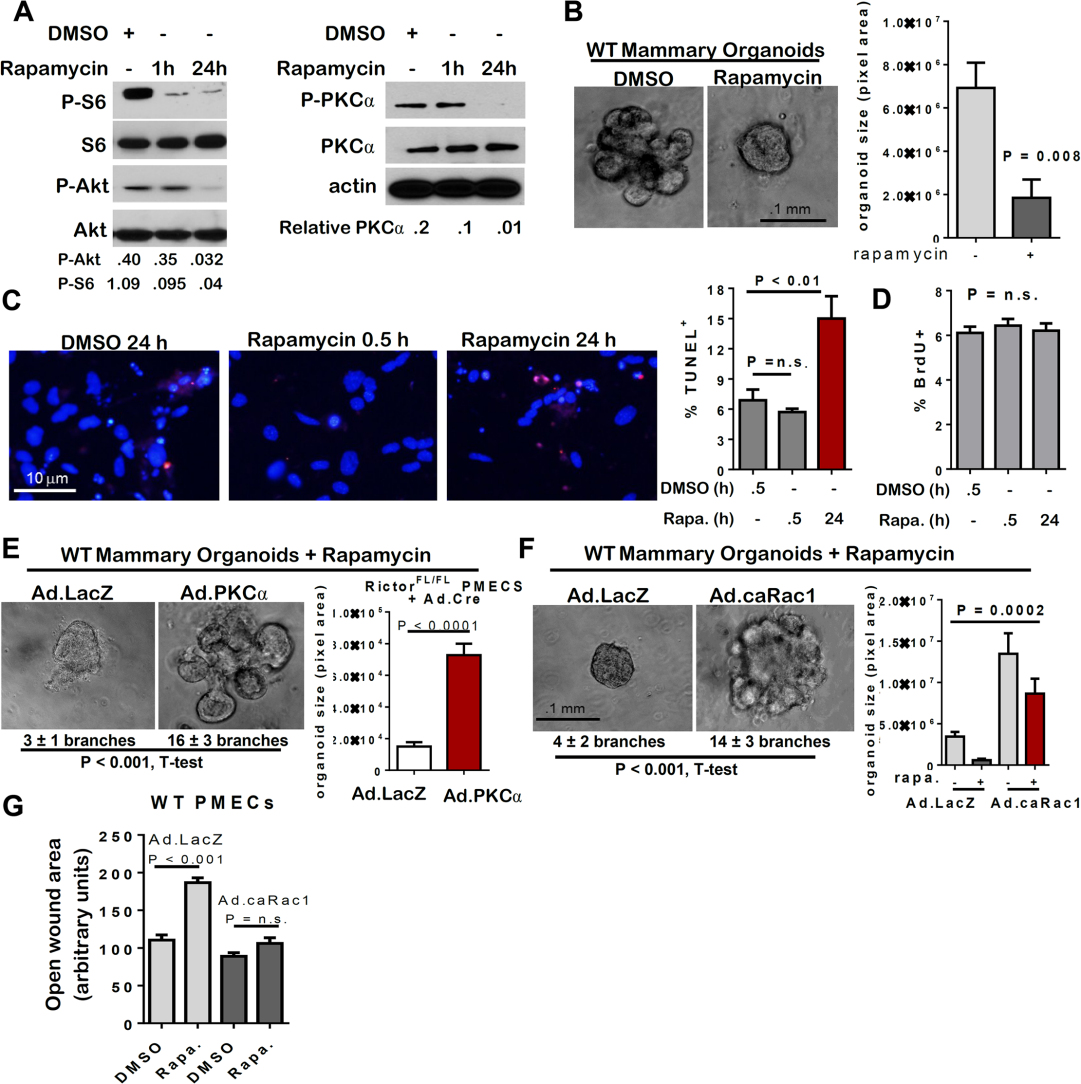
mTOR inhibition with rapamycin decreases MEC survival and branching. **A-G.** *WT* PMECs and organoids were cultured in DMSO vehicle or rapamycin. PMECs were cultured 0, 0.5, or 24 hours. Organoids were cultured 10 days in Matrigel in the presence of DMSO or rapamycin. **A**. Western analysis of PMEC lysates. Quantitation was performed using Image J software and numbers represent P-Akt, P-S6, or P-PKC-alpha bands normalized to total Akt, S6, or PKC-alpha levels. **B**. *WT* organoids photographed after 10 days in Matrigel culture. Average organoid size scored as average pixel area ± S.D., Student’s T-test, N = 6 epithelial isolates, each analyzed in triplicate. **C**. TUNEL analysis of PMECs. Average percent TUNEL+ nuclei per total PMEC nuclei ± S.D. is shown, Student’s T-test. N = 3 independent cell isolates, analyzed in duplicate. **D.** PMECs were labeled with BrdU after initial pre-treatments with rapamycin for 0.5 hours or 24 hours. Average percent BrdU+ nuclei per total nuclei ± S.D. is shown, One-way ANOVA, N = 3 independent cell isolates, analyzed in triplicate. **E-F.** Organoids were infected with Ad.LacZ or Ad.PKC-alpha (panel E) or Ad.caRac1 (panel F) prior to embedding in Matrigel plus rapamycin or DMSO. Average number of branches/colony is shown below each image. Average organoid size scored as average pixel area ± S.D., Student’s T-test (E) and one way ANOVA (F), N = 6 epithelial isolates, each analyzed in triplicate. **G.** Confluent PMEC monolayers were scratch-wounded, cultured in rapamycin or DMSO, and imaged at 24 hours. Total wounded area remaining after 24 hours was measured. Values shown are the average wound area remaining ± S.D. N = 3 independent cell isolates, analyzed in triplicate.

Rapamycin is a pharmacologic inhibitor of mTOR originally thought to preferentially inhibit mTORC1 over mTORC2. However, sustained rapamycin treatment impairs both mTORC1 and mTORC2 in a cell type-dependent manner [25–28]. Consistent with this idea, acute rapamycin treatment for 1 hour (1 h) decreased P-S6 (an mTORC1 effector) but not P-Akt (an mTORC2 effector), whereas sustained rapamycin treatment (24 h) decreased both P-S6 and P-Akt S473 (**Fig 6A**). Rapamycin treatment for 10 days significantly decreased branching morphogenesis and organoid size in *WT* organoids (**Fig 6B**). Although PMEC survival was not affected by acute rapamycin treatment, cell death increased after 24 h with rapamycin treatment (**Fig 6C**). Proliferation of *WT* PMECs, as measured by BrdU incorporation, was unaffected by acute (30 min) or sustained (24 h) pre-treatment with rapamycin (**Fig 6D**). The effects of sustained rapamycin treatment, including reduced MEC survival, branching morphogenesis formation, and diminished organoid size were similar to the effects achieved by Rictor ablation in MECs. Also similar to what was seen with Rictor-deficient MECs, the phenotypic effects of rapamycin treatment were rescued by Ad.PKC-alpha (**Fig 6E**) and Ad.caRac1 (**Fig 6F**), including rescue of branching morphogenesis and colony size. Ad.caRac1 also rescued rapamycin-mediated inhibition of cell motility (**Figs 6G and S5**). Because the mTOR inhibitor rapamycin impairs mTORC1 and mTORC2, and recapitulates the morphological and molecular effects of Rictor ablation in MECs, these results suggest that Rictor is acting in complex with mTOR to regulate MEC survival, motility, and branching morphogenesis, supporting a role for mTORC2 in the developing mammary gland. However, these findings do not rule out the contribution of mTORC1 to mTOR-mediated mammary morphogenesis.

### Raptor/mTORC1 loss produces relatively mild defects in mammary branching morphogenesis and epithelial growth *in vivo* and *ex vivo*

**Fig 7:**
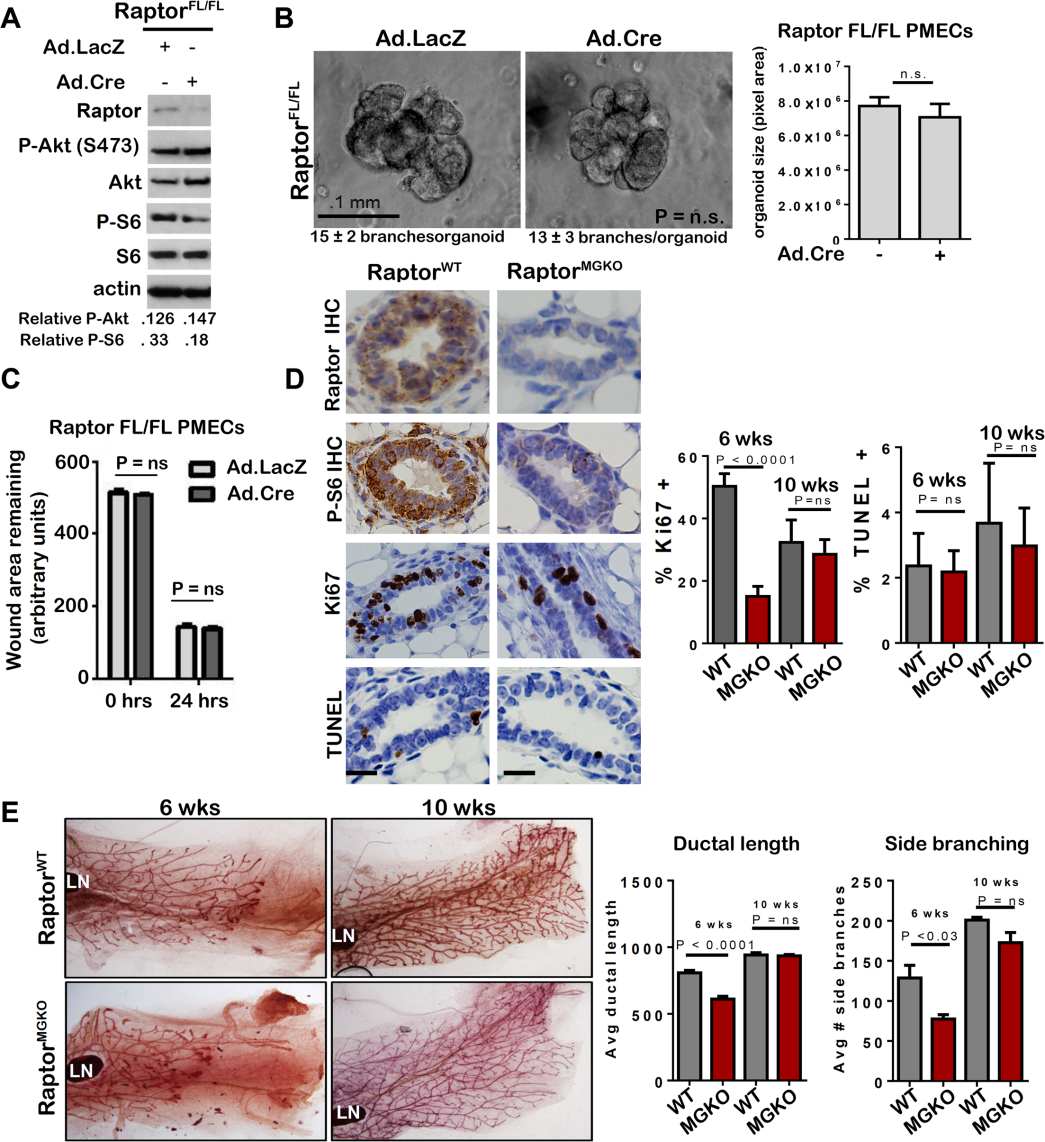
Unlike mTORC2, mTORC1 is dispensable for MEC survival and branching morphogenesis. **A-C.** *Raptor* ^*FL/FL*^ PMECs and organoids were infected with Ad.Cre and Ad.LacZ, and cultured 10 days. **A.** Western analysis of PMECs cultured in the absence of serum. Quantitation was performed using Image J software and numbers represent P-Akt or P-S6 bands normalized to total Akt or S6 levels. **B.** Organoids were infected with Ad.Cre or Ad.LacZ photographed after 10 days in Matrigel culture. Representative images are shown. Average number of branches/organoid for each group is shown below the image. Average organoid size (pixels) ± S.D. is shown, Student’s T-test. N = 6 independent organoid isolates, analyzed in triplicate**. C.** PMECs from *Raptor* ^*Fl/FL*^ mice were infected with Ad.LacZ or Ad.Cre, grown to confluence and scratch-wounded. Monolayers were imaged. Total wound area remaining was measured 24 hours after wounding. Values shown are the avg ± S.D. N = 6 per time point, Student’s T-test. **D-E.** Mammary glands from virgin female Raptor^WT^ and Raptor^MGKO^ at 6 and 10 weeks of age were analyzed. N = 10 mice per genotype at each time point. Statistical analysis performed with Student’s T-test. **D.** IHC for Raptor, P-S6, Ki67+, and TUNEL+ nuclei in mammary glands of 6-week old mice. Representative images are shown. Scale bars = 50 microns. Average percent Ki67+ nuclei and TUNEL+ nuclei (± S.D) per total epithelial nuclei was determined **E.** Whole mount hematoxylin staining of mammary glands. Representative images are shown. LN = lymph node. The ductal length beyond the mammary lymph node was measured in whole mounted mammary glands. Average length (in microns) ± S.D. is shown. The number of T-shaped side branches was enumerated in whole mounted mammary glands. Values shown represent average number of side branches ± S.D.

To understand how mTORC1 participates in mammary morphogenesis, we infected PMECs harvested from female *Raptor* ^*FL/FL*^ mice [29] with Ad.Cre. Western analysis confirmed loss of Raptor and decreased P-S6 in serum-deprived cells (**Fig 7A**). However, P-Akt S473 was unaffected by Raptor ablation, confirming that genetic ablation of Raptor causes selective inhibition of mTORC1, while Rictor ablation inhibits mTORC2. *Raptor* ^*FL/FL*^ mammary organoids infected with Ad.LacZ formed multi-branched colonies, as expected (**Fig 7B**). Surprisingly, infection with Ad.Cre did not affect branching morphogenesis in *Raptor* ^*FL/FL*^ organoids, the number of branches per organoid, or colony size (**Fig 7B**). Additionally, Raptor ablation had no significant impact on PMEC migration in wound healing assays **(Fig 7C).**

*Raptor* ^*FL/FL*^ *MMTV-Cre* (*Raptor* ^*MGKO*^) mice were used to assess the impact of Raptor ablation on mammary morphogenesis *in vivo*. IHC detected Raptor and the mTORC1 effector P-S6 in *Raptor* ^*WT*^ mammary glands at 10 weeks of age but did not detect P-S6 in age-matched *Raptor* ^*MGKO*^ mice (**Fig 7D**). Western analysis of whole mammary lysates from 10 week-old mice confirmed loss of Raptor **(S6A Fig).** Immunofluorescent (IF) staining for cytokeratin (CK)-8 and CK14, molecular markers of luminal and myoepithelial MECs, respectively, confirmed that Raptor loss did not affect the relative spatial organization of luminal and myoepithelial MECs **(S6B Fig–upper panel).** Additionally, no alterations in localization or staining pattern of ZO-1 were observed **(S6B Fig–lower panel).** Proliferation, as measured by IHC for Ki67, was significantly decreased in *Raptor* ^*MGKO*^ ducts in 6-week old mice, but not in TEBs **(S6C Fig)**. By 10 weeks, however, proliferation in ducts had recovered to levels seen in *Raptor* ^*WT*^ (**Fig 7D**). TUNEL analysis revealed similar ratios of TUNEL+ MECs in *Raptor* ^*MGKO*^ and *Raptor* ^*WT*^ samples harvested from 6 and 10 week old animals **(Fig 7D)**. Consistent with these observations, only mild defects in side branching and ductal length were found in mammary glands from 6-week old *Raptor* ^*MGKO*^ mice (**Fig 7E**), and these were resolved by 10 weeks of age.

Taken together, these results demonstrate that mTOR uses Rictor to activate PKCα/Rac1-dependent survival, motility, and branching morphogenesis in the mammary epithelium and that Rictor does not rely fully on Akt signaling to promote ductal morphogenesis in the breast.

## Discussion

Postnatal mammary epithelial morphogenesis requires precise coordination of cell proliferation, apoptosis, differentiation, and motility in order to turn rudimentary epithelial buds into an organized, branched ductal network permeating the entire mammary fat pad by the end of puberty [1, 2]. mTOR is a central regulator of proliferation, apoptosis, differentiation, and motility, integrating numerous upstream signals to generate the desired biological outcome. Therefore, we assessed how mTOR signaling contributes to mammary morphogenesis. We found that pharmacologic mTOR inhibition reduced the size and branching complexity of mammary organoids in culture, phenotypes recapitulated by mTORC2 loss of function via *Rictor* ablation, but not upon mTORC1 inhibition through *Raptor* ablation. We also observed a disorganized epithelial architecture and stromal thickening around TEB upon tissue-specific Rictor ablation. The MMTV-Cre model has been reported to be leaky, leading to expression in tissues other than luminal mammary epithelium [30] thus it is possible that some of these defects may be due to loss of Rictor in stromal components. Alternatively, changes in basal epithelium may be a secondary effect of luminal cell misolocalization in the absence of Rictor, or Rictor expression in the luminal compartment may regulate expression and function of mTOR signaling intermediates in the basal cell layer through an indirect, juxtacrine signaling mechanism. We are actively investigating the role of Rictor/mTORC2 in luminal versus basal epithelium in our ongoing research. As our epithelial branching and survival phenotypes were recapitulated in the *ex vivo* stroma-free organoid culture model, however, it is likely that the effects on stroma are, at least in part, secondary to the loss of Rictor in epithelium.

Genetic inhibition of mTORC2 also reduced ductal branching and lengthening *in vivo*, diminished P-Akt and P-PKC-alpha, and impaired activation of the GTPase Rac1. Akt restoration only modestly enhanced branching morphogenesis in Rictor-deficient mammary organoids and was not sufficient to rescue cell survival or PMEC invasion through Matrigel. However, Akt inhibition did decrease organoid branching and colony size suggesting that Akt provides a critical signal in growth control, but is not sufficient to drive branching morphogenesis in the absence of Rictor. This is consistent with the data from our analysis of transplanted Rictor-deficient/Akt^Myr^ expressing MEC *in vivo* and with the phenotype of Akt1 deletion, which did not affect mammary epithelial cell differentiation but did impair lactation [31]. Deletion of Akt1 and one allele of Akt2 enhanced this defect [32, 33]. Moreover, Akt activation did not completely inhibit luminal apoptosis during MCF10A acinar morphogenesis in culture [34], suggesting that other factors also regulate cell survival during normal mammary epithelial development. In contrast to Akt, restoration of PKC-alpha signaling to Rac1, or Rac1 activation independently of upstream signals, fully rescued all phenotypes resulting from Rictor loss in culture and in transplanted Rictor-deficient MEC *in vivo*, suggesting that Rictor-dependent mTORC2 is essential for PKC-alpha-Rac1 signaling to drive mammary morphogenesis. While not directly tested here, at least one additional study has elucidated mechanisms downstream of Rac1 that can control cell survival. One report using lymphoma cells demonstrated direct inhibition of apoptosis through Rac1-stimulated phosphorylation of the Bcl-2 family member, Bad, which occurred in an Akt-independent manner [35]. We observed a modest decrease in cell viability upon prolonged treatment with the Rac1 inhibitor in organoid culture coupled with the decreased branch extension, consistent with previous studies that also reported regulation of branching initiation and extension via PI3K-mediated Akt and Rac1, respectively [36].

Interestingly, levels of mTORC1 target P-S6 are elevated in MEC upon Rictor loss relative to controls. This could reflect shift of mTOR kinase to complex 1 in the absence of a stable mTORC2 complex. It will be of great interest to track mTOR kinase association with the two complexes over the course of mammary epithelial development to better understand its functions. Activation of the Akt signaling pathway upon mTOR inhibition via a negative feedback loop has been observed in many cell types, including breast cancer cell lines (Reviewed in [37]). In our study, rapamycin preferentially inhibited mTORC1 upon acute treatment (e.g. reduction in P-S6 without affecting P-Akt-S473 levels) and as prolonged treatment inhibited both complexes (e.g. reduction in both P-S6 and P-Akt-S473). These data are consistent with the observation that rapamycin is an effective inhibitor for activity of both complexes in many cell types [5], including MECs. The differences in response to rapamycin between normal MECs and breast cancer cell lines could be due to differences in insulin-like growth factor receptors (IGFRs), which are expressed at higher levels in cancer cells and mediate feedback to Akt upon mTOR inhibition (Reviewed in [37–39]).

Given the known roles of mTORC1 in cell growth, metabolism, and protein and lipid synthesis [3], it was surprising that Raptor loss produced only a transient delay in ductal lengthening. It is possible that other signaling pathways may compensate for loss of mTORC1 function in Raptor-deficient mammary epithelium, such as RSK-mediated activation of S6 [40]; [41]. However, we observed similar decreases in cellular proliferation in the absence of Raptor and Rictor expression at 6 weeks that recovered by 10 weeks, suggesting that MEC proliferation may rely on both mTORC1 and mTORC2. Decreased MEC proliferation upon genetic mTORC1 ablation is consistent with other reports of rapamycin-mediated cell growth inhibition in lactating mouse mammary explants, in lactating mice, and in milk-producing HC11 cells. Based on these previous studies, it will be important to determine the effects of Raptor and Rictor ablation on growth, differentiation, and milk production in alveolar mammary epithelium during pregnancy and lactation *in vivo*.

The PI3-kinase (PI3K)/mTOR pathway is aberrantly activated up to 60% of clinical breast cancers, facilitating tumor cell growth, survival, metabolism, and invasion [42, 43]. Moreover, increased PI3K activity in *MMTV-Cre/PTEN* ^*FL/FL*^ mice increases mammary epithelial branching and decreases apoptosis during pubertal development [44], suggesting that PI3K signaling is important in branching and survival in the breast. This idea is consistent with the phenotype produced by *MMTV-Cre*-driven Rictor loss, in which loss of a PI3K pathway mediator produces decreased branching and survival. While inhibitors of mTORC1 show limited clinical efficacy as single agents, anti-PI3K agents combined with dual mTORC1/2 inhibitors appear to be more effective [45–48], underscoring the clinical relevance of mTORC2 in breast cancer. Importantly, these recent clinical observations parallel the data shown here demonstrating that mTORC2 inhibition due to either sustained rapamycin treatment or to Rictor deletion profoundly affected the complex series of events driving mammary morphogenesis, and these mTORC2-dependent processes occur in a manner unique and separable from mTORC1. Interestingly, preferential targeting of mTORC2 versus mTORC1 reduced breast cancer cell motility and survival in culture and *in vivo* [18, 49], and Rictor knockdown suppressed anchorage-independent growth of MCF7 breast tumor cells [50]. Although at least one report suggests elevated Rictor levels correlate with higher overall and recurrence-free survival [51], Rictor overexpression was observed in clinical invasive breast cancer specimens relative to normal breast tissue, as well as in lymph node metastases [18], supporting the clinical relevance of mTORC2 in invasive breast cancer. Given our findings that Rictor/mTORC2 is required in the normal mammary epithelium for PKC-alpha-Rac1 activation which drives MEC survival, motility, and invasion, it will be interesting to determine if the mTORC2-PKC-alpha-Rac signaling axis is used by breast cancer cells to drive metastasis. If so, mTORC2-specific targeting or PKC-alpha inhibition could represent potential therapeutic strategies to limit metastatic spread of breast tumors, and to limit survival of disseminated tumor cells.

Although data shown herein are the first demonstration of mTORC2-mediated regulation of normal MEC migration and invasion, several lines of evidence suggest that cancer cells exploit Rictor-dependent signaling pathways to facilitate invasion and metastasis. For example, siRNA-mediated Rictor knockdown inhibited MCF7 and MDA-MB-231 breast cancer cell migration [18, 49]. Rictor knockdown inhibited transforming growth factor beta (TGF-beta)-mediated epithelial-to-mesenchymal transition (EMT) in breast cancer lines [52]. In contrast to our findings that untransformed MECs use Rictor to activate PKC-alpha and Rac1-mediated invasion, breast cancer cells used Rictor to drive motility through protein kinase C-zeta (PKC-zeta; [18]), integrin-linked kinase (ILK; [52]) and Akt [49]. Although Akt phosphorylation at S473 required Rictor/mTORC2 in primary MECs, restoring Akt function was not sufficient to rescue survival, motility, or branching morphogenesis in the absence of Rictor. Restoration of Rac1 activity, an essential regulator of mammary epithelial branching morphogenesis [16, 53] and a downstream effector of mTORC2 and PKC-alpha, rescued survival and migration defects induced by genetic mTORC2 inhibition. While not specifically linked to Rictor in breast cancer cells, Rac1-mediated invasion and metastasis of breast cancer cells has been reported previously [54–56]. Together, these data suggest that Rictor/mTORC2-dependent Rac signaling could promote breast cancer invasion, paralleling its function normal MEC branching morphogenesis. It is possible that breast cancer cells can engage multiple pathways (PKC-zeta, ILK, Akt, Rac, and others) to regulate tumor cell metastasis, and it is interesting to speculate that Rictor may lie at the intersection of each of these pathways.

In summary, our data demonstrate distinct, non-overlapping functions of mTORC1and mTORC2 in post-natal mammary morphogenesis. Whereas Raptor-dependent mTORC1 signaling regulates proliferation, Rictor-dependent mTORC2 is essential for cell survival, cell junctions, motility, and branching morphogenesis. These findings underscore the importance of understanding the distinct roles for mTORC1 and mTORC2 in normal physiology of the breast and in breast cancer in order to intelligently develop and administer mTOR-directed therapies.

## Materials and Methods

### Mice

All animals were housed under pathogen-free conditions, and experiments were performed in accordance with AAALAC guidelines and with Vanderbilt University Institutional Animal Care and Use Committee approval. *Rictor* ^*FL/FL*^ mice (C57BL/6) were kindly provided by Dr. Mark Magnuson (Vanderbilt University) and have been previously described [14]. *Raptor* ^*FL/FL*^ mice ([29], C57BL/6) were purchased from the Jackson Laboratories (Bar Harbor, ME). *MMTV-Cre* mice ([13] FVB) were purchased from the Jackson Laboratories. All analyses were performed on age-matched siblings resulting from F1 (1:1, FVB:C57BL/6) intercrosses.

### PMEC and organoid cell culture

Primary mammary organoids were generated from freshly collected, partially disaggregated mouse mammary glands using a modification of previously described methods [16]. Primary mouse mammary epithelial cells (PMECs) were harvested as described previously [57]. Organoids were immediately embedded in growth factor reduced Matrigel (BD Bioscience) at 50 organoids/100 microliters. Once polymerized, Matrigel-embedded cultures were overlain with Growth Media [DMEM:F12 supplemented with 5 micrograms/ml porcine insulin (Sigma-Aldrich), 10 picograms/ml each estrogen and progesterone (Sigma-Aldrich), 5 nanograms/ml human epidermal growth factor (R&D Systems), 100 I.U./ml penicillin-streptomycin (Life Technologies)]. PMEC were maintained in Growth Media. For some experiments, cells were maintained for 24 hour in Starvation Media [Growth Media supplemented with penicillin-streptomycin only] or treated with Fibroblast-Conditioned Media (DMEM:F12 supplemented 100 I.U./ml penicillin-streptomycin cultured with mouse mammary fibroblasts for 48 hours and passed through a 0.2 micron filter) for wound closure migration studies. Rapamycin (Sigma-Aldrich, 20 nanomolar), InSolution Rac1 inhibitor (Calbiochem/Millipore, 20 micromolar), and adenoviral particles (Ad.Cre, Ad.LacZ, Ad.caRac1, Ad.Akt^myr^, and Ad.PKC-alpha, Vector Biolabs) were purchased. Freshly collected organoids were incubated with adenoviral particles (5 X 10^8^ particle forming units/ml) with constant rocking for 3–5 hours at 37°C, washed, and embedded in Matrigel. We analyzed 20–30 independent organoids isolated from 5–6 independent mice in 2–3 experiments for each condition.

Morphogenesis in organoids was scored by counting the number of branches/organoid in 10 or more organoids/culture condition. For structures that appeared more spherical and less branched (e.g. cultures treated with Ad.Cre or inhibitors), we counted bifrucations and/or small protrusions from ball-shaped structures as branches in order to be as rigorous and conservative in our quantifications as possible. Organoid size was scored using NIH Image J software to quantify pixel area in 10 or more organoids/culture condition.

### MCF10A cell culture

MCF10A and MCF10A Rictor^ZFN^ were purchased from Sigma-Aldrich and cultured in Growth Medium [DMEM:F12 supplemented with 5% Horse Serum (Life Technologies), 10 μg/ml porcine insulin (Sigma-Aldrich), 20 nanograms/ml human epidermal growth factor (R&D Systems), 10 nanograms/ml cholera toxin (Sigma-Aldrich), 100 micrograms/ml hydrocortisone (Sigma-Aldrich), 100 I.U./ml penicillin-streptomycin (Life Technologies)]. For some experiments, cells were maintained for 24 hour in Starvation Media [Growth Media without serum or EGF] prior to stimulation and/or analysis. PKC-alpha inhibitor GO6976 (Sigma-Aldrich, 2 nm) and adenoviral particles (Ad.RFP and Ad.PKC-alpha, Vector Biolabs) were purchased. Cells were incubated with adenoviral particles (5 X 10^8^ particle forming units/ml) for 3–5 hours at 37°C and cells were allowed to recover for 48 hours prior to experimental analysis.

### Immunofluorescence

Matrigel-emdedded organoids cultured on coverslips were fixed 8 minutes in 1:1 methanol:acetone at -20°C, permeabilized in 0.5% Triton-X 100/PBS for 10 min, blocked [130 millimolar NaCl, 7 millimolar Na_2_HPO_4_, 3.5 millimolar NaH_2_PO_4_, 7.7 millimolar NaN_3_, 0.1% bovine serum albumin, 0.2% Triton-X 100, 0.05% Tween-20] and stained with rabbit anti-pan-cytokeratin (Santa Cruz Biotechnology, 1:100) and AF621-goat anti-rabbit (1:100), counterstained with TO-PRO-3 Iodide (Invitrogen), and imaged using the Vanderbilt Cell Imaging Shared Resource Zeiss LSM 510 confocal microscope and LSM Image Browser software. For E-cadherin staining, the primary antibody used was anti-E-cadherin (BD Transduction Laboratories) and visualized with anti-mouse Alexa 594 secondary antibody (Invitrogen, Molecular Probes). Confocal images of 3D structures were visualized using an LSM 510 META inverted confocal microscope with a 20X/0.75 plan apochromat objective.

### Western blotting

Cells and tissues were homogenized in ice-cold lysis buffer [50 millimolar Tris pH 7.4, 100 millimolar NaF, 120 millimolar NaCl, 0.5% NP-40, 100 micromolar Na_3_VO_4_, 1X protease inhibitor cocktail (Roche)], sonicated 10 seconds, and cleared by centrifugation at 4°C, 13,000 x *g* for 5 min. Protein concentration was determined using BCA (Pierce). Proteins were separated by SDS-PAGE, transferred to nitrocellulose membranes, blocked in 3% gelatin in TBS-T [Tris-buffered saline, 0.1% Tween-20), incubated in primary antibody overnight and in HRP-conjugated anti-rabbit or anti-mouse for 1 hour, and developed using ECL substrate (Pierce). Antibodies used: alpha-actin (Sigma-Aldrich; 1:10,000); AKT and S473 P-Akt (Cell Signaling; 1:2,000 and 1:500, respectively); S6 and P-S6 (Cell Signaling; 1:1,000); Rictor (Santa Cruz; 1:250); Raptor (Cell Signaling; 1:500); Rab11 (Cell Signaling; 1:1,000); PKC-alpha and T638/641 P-PKC-alpha (Cell Signaling; 1:2,000); Rac (BD Transduction; 1:200). GST-Pak-PBD effector pulldown assays were performed using reagents from Millipore as per manufacturer’s protocol.

### Histological analysis

Mammary glands were whole-mounted on slides, cleared of adipose, and stained with hematoxylin as described previously [57]. Sections (5 micron) were stained with hematoxylin and eosin. *In situ* TUNEL analysis was performed on paraffin-embedded sections using the ApopTag kit (Calbiochem). IHC on paraffin-embedded sections was performed as described previously [58] using: Ki67 (Santa Cruz Biotechnologies), P-S6 (Cell Signaling Technologies); P-Akt S473 (Cell Signaling Technologies); Rictor (Santa Cruz), E-cadherin (Transduction Labs). Immunodetection was performed using the Vectastain kit (Vector Laboratories), AF488-conjugated anti-rabbit, or AF621-conjugated anti-mouse (Life Technologies), according to the manufacturer’s directions.

### *In situ* Rac-GTP assay

Methanol-fixed PMECs were probed 1 hour with GST-PBD (Millipore) diluted 1:50 in PBS. GST (lacking PBD) was used as a negative control. Samples were washed then probed with AF488-conjugated anti-GST (1:100), stained with DAPI or AF621-phalloidin, and mounted.

### Transwell migration and wound closure assays

MECs (10^5^) were added to upper chambers of Matrigel-coated transwells in starvation medium and incubated 5 hours to score migration in response to 10% serum-containing medium in the lower chamber. Filters were swabbed and stained with 0.1% crystal violet, [59] and cells on the lower surface were counted. For wound closure, 50,000 MECs were plated on Matrigel-coated 24 well plates, grown to confluence, serum-starved for 24 hours, and wounded with a P200 pipette tip. Migration in response to mammary fibroblast conditioned medium [60] was scored by measuring the [width of the wound area at 24 hours] ÷ [width of the wound area at 0 hours] as described previously [61].

### Microscope image acquisition

Mammary gland whole-mounts and transwell filters were imaged with Olympus SZX12 Inverted Microscope. Slides were imaged with Olympus BX60 Stereo Microscope. Organoids, annexin V-FITC-staining, and wound closure assays were imaged with Olympus IX71 Inverted Microscope. All images were acquired by Olympus DP 72 Digital Camera and CellSens software at ambient temperature.

### Ethics statement

All animals were housed under pathogen-free conditions, and experiments were performed in accordance with AAALAC guidelines and with Vanderbilt University Institutional Animal Care and Use Committee approval. The laboratory animal care program of Vanderbilt University (PHS Assurance #A3227-01) has been accredited by AAALAC International since 1967 (File #000020). The AAALAC Council on Accreditation's most recent review of VU's program was done in 2011 and resulted in "Continued Full Accreditation.” Isofluorane was used for anesthesia, as well as euthanasia. For human euthanasia, cervical dislocation was used following isofluorane overdose.

## Supporting Information

S1 Fig Loss of Rictor disrupts mammary branching morphogenesis in vivo. **A.** Hematoxylin stained whole mount preparations from 6 week old Rictor^WT^ mice and Rictor^MGKO^ mice. High magnification panels show irregular ductal tracts and increased staining density in Rictor^MGKO^ samples, indicative of multiple cell layers. Data are a representation of 11 independent animals/genotype. **B.** Hematoxylin stained whole mount preparations from 10 week old wild-type Rictor^WT^ mice and Rictor^MGKO^ mice show sustained length defects in the ductal tracts of mammary glands lacking Rictor. **C.** IHC for Ki67 or TUNEL in TEBs from 6 week old wild-type Rictor^WT^ mice and Rictor^MGKO^ mice. Average percent Ki67 and TUNEL+ nuclei (± S.D.) was determined. **D.** Confocal analysis of primary organoids (PMOs) stained for E-cadherin (red) revealed multiple cell layers in acinar structures and poor lumen formation in Rictor-deficient (*Rictor* ^*FL/FL*^ organoids infected with Ad.Cre) PMOs relative to control PMOs (*Rictor* ^*FL/FL*^ organoids infected with Ad.LacZ), consistent with the phenotype in Rictor^MGKO^ epithelium *in vivo*.
(TIF)

S2 Fig Loss of Rictor impairs branching and lumen formation in organoid culture. Primary organoids (PMOs) were isolated from *Rictor* ^*FL/FL*^ mice and infected with Ad.GFP or Ad.Cre. **A.** Fluorescent imaging of organoids 24 hours post-infection. **B.** Confocal analysis of WT organoids infected with Ad.GFP. **C.** Organoids were fixed 10 days post-infection and subjected to immunofluorescent staining using a pan-cytokeratin antibody (red) to confirm epithelial identity and ToPro-3 nuclear marker (blue). While Ad.LacZ-infected *Rictor* ^*FL/FL*^ organoids formed hollow lumens surrounded by an organized epithelial layer, Ad.Cre-infected *Rictor* ^*FL/FL*^ organoids remained rounded and disorganized. Data are a representation of 3 independent organoid isolates. **D.** MCF10A parental or MCF10A-Rictor^ZFN^ cells were assessed for invasion through Matrigel-coated transwell filters. The cells remaining in the upper chamber after 24 hours were fixed and stained with either DAPI (blue) or Annexin V-FITC (green). The average percent viable cells (± S.D.) left in the upper chamber was quantitated using Image J, Student’s T-test.
(TIF)

S3 Fig Akt activation is insufficient to rescue Rictor-deficient MEC survival, branching and invasion. **A-C.** PMECs and organoids from *Rictor* ^*FL/FL*^ mice were coinfected with Ad.Cre and either Ad.LacZ or Ad.Akt^DD^. **A.** Western analysis of PMEC lysates. **B.** Organoids photographed after 10 days in Matrigel culture. Average number of branches/organoid ± S.D. (right panel) is shown. N = 7 independent organoid isolates, analyzed in triplicate. Midline values indicate average, whiskers indicate S.D., Student’s T-test. **C.** Colony size of organoids (± S.D.) measured in pixel area. N = 7 independent organoid isolates, analyzed in triplicate, Student’s T-test.
(TIF)

S4 Fig Rictor-mediated PKC-alpha activation controls acinar formation and motility in human and mouse mammary epithelial cells. **A.** Immunofluorescent detection of P-PKC-alpha in mammary gland sections from 6 week old virgin *Rictor* ^*WT*^ and *Rictor* ^*MGKO*^ mice. **B.** *In situ* detection of GTP-only control (no PBD) via IF detection of GST-PBD (red; nuclei stained with DAPI, blue) on mammary gland sections from 6-week old mice. **C.** *WT* PMECs cultured ± Rac inhibitor or *Rictor* ^*FL/FL*^ PMECs infected with Ad.LacZ or Ad.Cre were assessed for GTP-bound Rac via IF detection of GST-PBD (red; nuclei stained with DAPI, blue). **D.** *Rictor* ^*FL/FL*^ PMECs infected with Ad.Cre or Ad.LacZ were probed with GST-PBD (green) and counterstained with phalloidin (red). Representative images are shown. **E.** *Rictor* ^*FL/FL*^ PMECs infected with Ad.Cre or Ad.LacZ (±Ad.caRac1) were analyzed for TUNEL. **F.** Whole mount analysis of endogenous mammary glands harvested from WT recipient mice.
(TIF)

S5 Fig mTOR inhibition with rapamycin decreases MEC survival, motility and branching, which are rescued by Rac activity. *WT* PMECs infected with Ad.LacZ or Ad.caRac1 were grown to confluence (3–5 days after infection) then wounded using a P200 pipette tip in the presence of DMSO or rapamycin. Monolayers were imaged after 24 hours and total wounded area (arbitrary units) remaining was measured at 24 hours after wounding. Values shown are the average ± S.D. N = 6 per time point.
(TIF)

S6 Fig Unlike mTORC2, mTORC1 is dispensable for MEC survival and acinar formation. **A.** Western analysis of whole mammary gland lysates from 10 week old mice. **B.** IF for CK8, CK14 and ZO-1 in mammary gland sections. **C.** IHC for Ki67 or TUNEL in TEBs from 6 week old wild-type *Raptor* ^*WT*^ mice and *Raptor* ^*MGKO*^ mice. Average percent Ki67+ and TUNEL+ nuclei (± S.D.) was determined, Student’s T-test.
(TIF)
