# Supplementary figures and images for: mTOR Directs Breast Morphogenesis through the PKC-alpha-Rac1 Signaling Axis
Source: PLoS Genet. 2015 Jul 1;11(7):e1005291. doi: 10.1371/journal.pgen.1005291 (PMC4488502; doi:10.1371/journal.pgen.1005291)

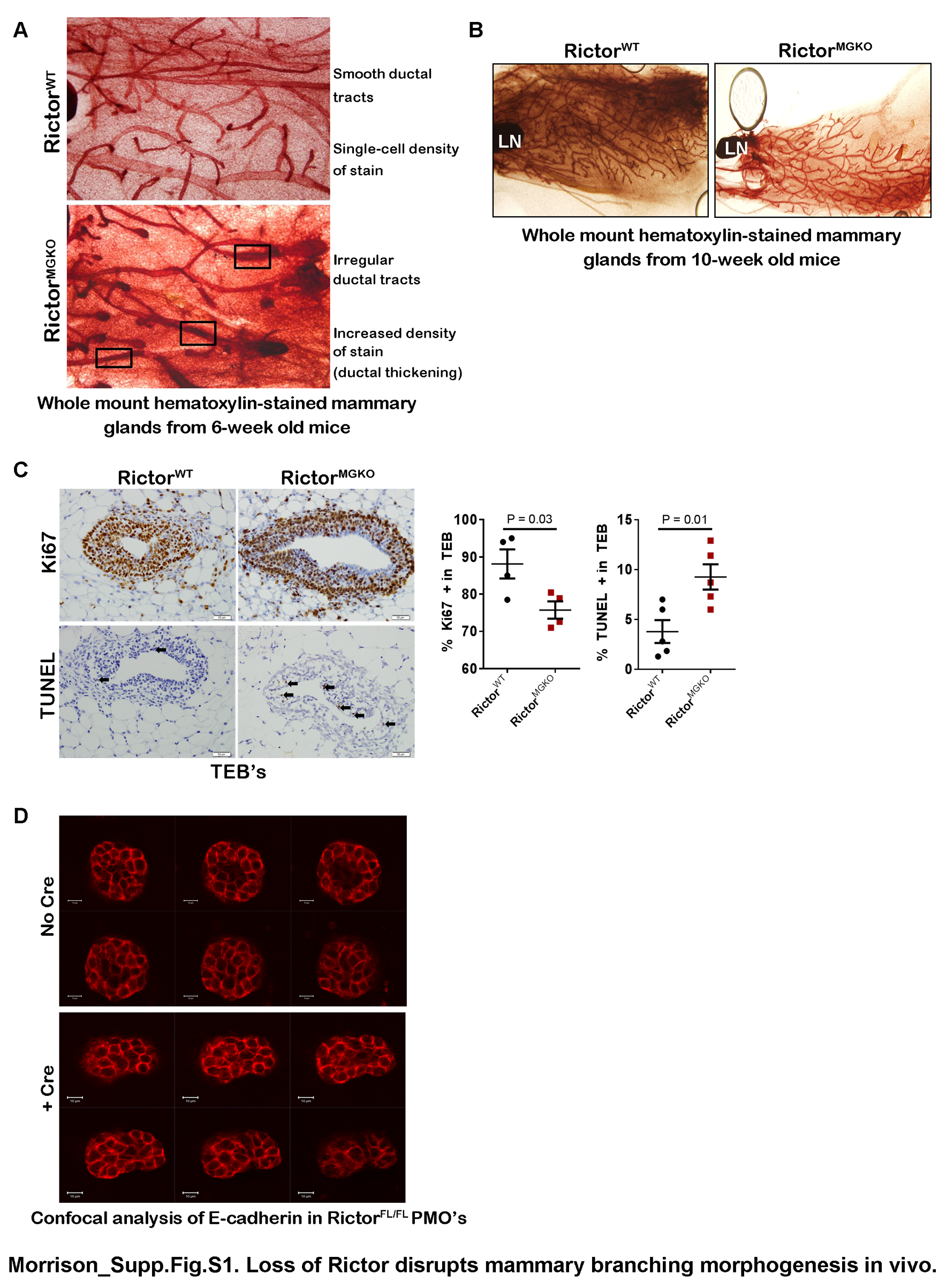

Supplement: S1 Fig — A. Hematoxylin stained whole mount preparations from 6 week old RictorWT mice and RictorMGKO mice. High magnification panels show irregular ductal tracts and increased staining density in RictorMGKO samples, indicative of multiple cell layers. Data are a representation of 11 independent animals/genotype. B. Hematoxylin stained whole mount preparations from 10 week old wild-type RictorWT mice and RictorMGKO mice show sustained length defects in the ductal tracts of mammary glands lacking Rictor. C. IHC for Ki67 or TUNEL in TEBs from 6 week old wild-type RictorWT mice and RictorMGKO mice. Average percent Ki67 and TUNEL+ nuclei (± S.D.) was determined. D. Confocal analysis of primary organoids (PMOs) stained for E-cadherin (red) revealed multiple cell layers in acinar structures and poor lumen formation in Rictor-deficient (Rictor FL/FL organoids infected with Ad.Cre) PMOs relative to control PMOs (Rictor FL/FL organoids infected with Ad.LacZ), consistent with the phenotype in RictorMGKO epithelium in vivo. (TIF) [file pgen.1005291.s001.tif]

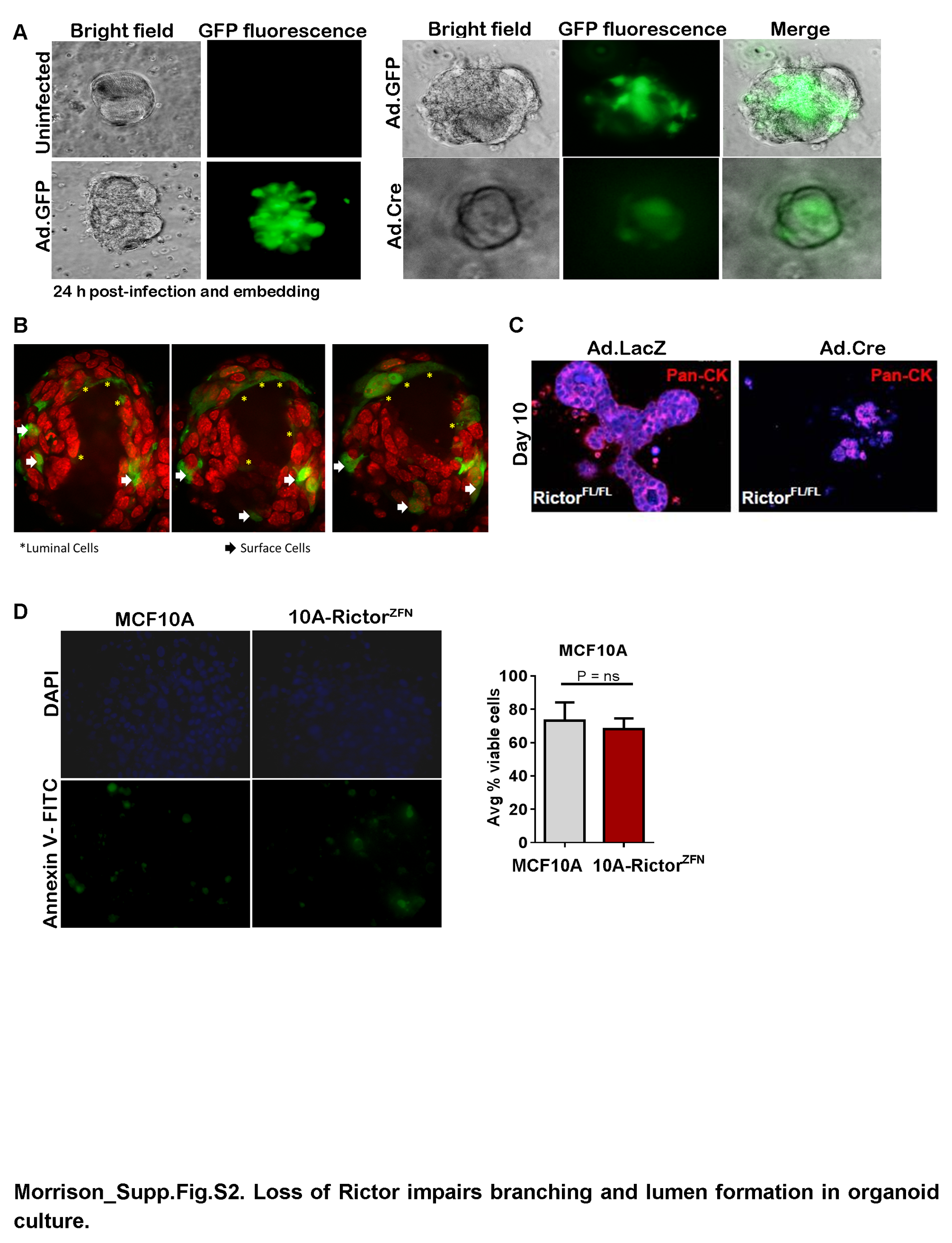

Supplement: S2 Fig — Primary organoids (PMOs) were isolated from Rictor FL/FL mice and infected with Ad.GFP or Ad.Cre. A. Fluorescent imaging of organoids 24 hours post-infection. B. Confocal analysis of WT organoids infected with Ad.GFP. C. Organoids were fixed 10 days post-infection and subjected to immunofluorescent staining using a pan-cytokeratin antibody (red) to confirm epithelial identity and ToPro-3 nuclear marker (blue). While Ad.LacZ-infected Rictor FL/FL organoids formed hollow lumens surrounded by an organized epithelial layer, Ad.Cre-infected Rictor FL/FL organoids remained rounded and disorganized. Data are a representation of 3 independent organoid isolates. D. MCF10A parental or MCF10A-RictorZFN cells were assessed for invasion through Matrigel-coated transwell filters. The cells remaining in the upper chamber after 24 hours were fixed and stained with either DAPI (blue) or Annexin V-FITC (green). The average percent viable cells (± S.D.) left in the upper chamber was quantitated using Image J, Student’s T-test. (TIF) [file pgen.1005291.s002.tif]

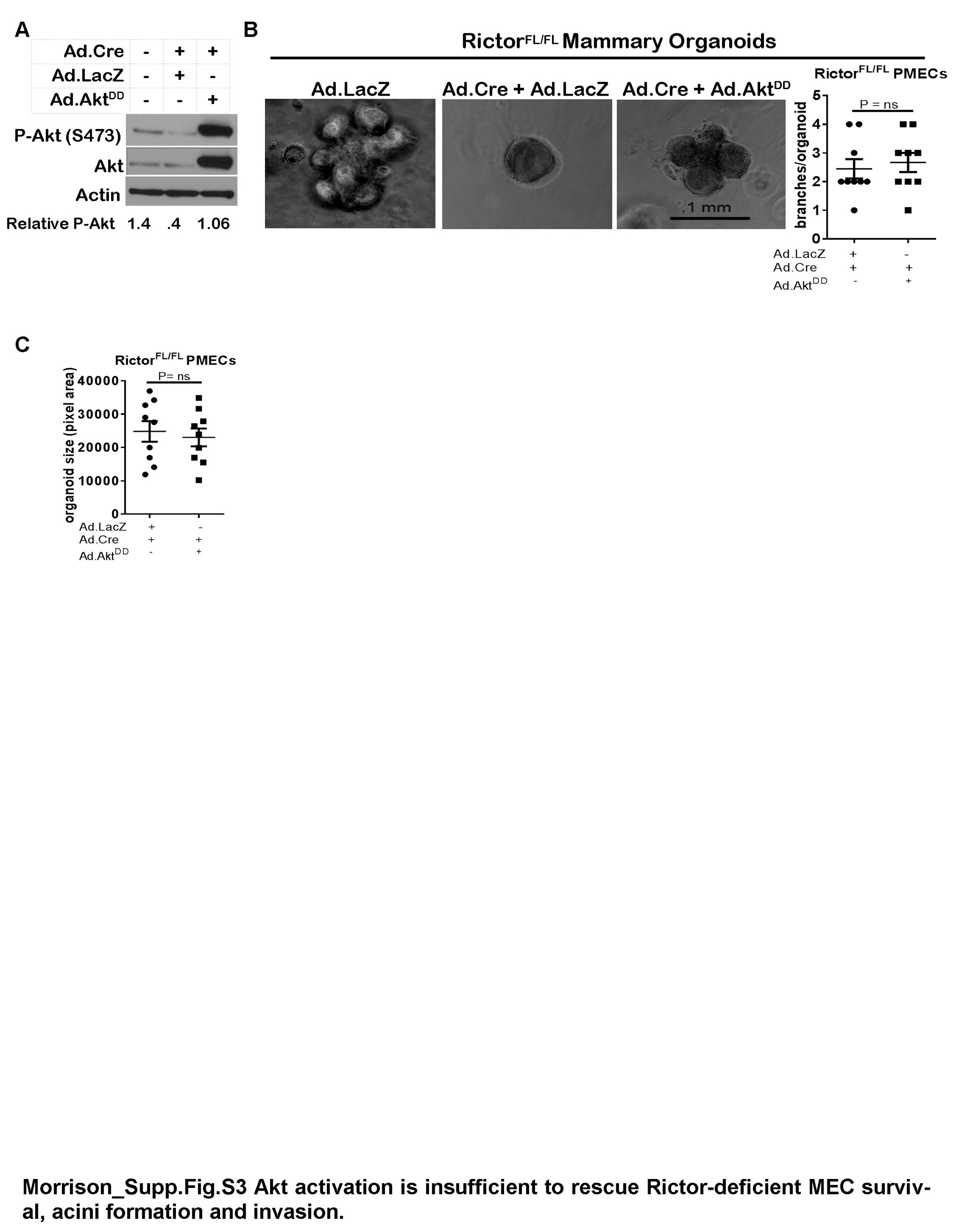

Supplement: S3 Fig — A-C. PMECs and organoids from Rictor FL/FL mice were coinfected with Ad.Cre and either Ad.LacZ or Ad.AktDD. A. Western analysis of PMEC lysates. B. Organoids photographed after 10 days in Matrigel culture. Average number of branches/organoid ± S.D. (right panel) is shown. N = 7 independent organoid isolates, analyzed in triplicate. Midline values indicate average, whiskers indicate S.D., Student’s T-test. C. Colony size of organoids (± S.D.) measured in pixel area. N = 7 independent organoid isolates, analyzed in triplicate, Student’s T-test. (TIF) [file pgen.1005291.s003.tif]

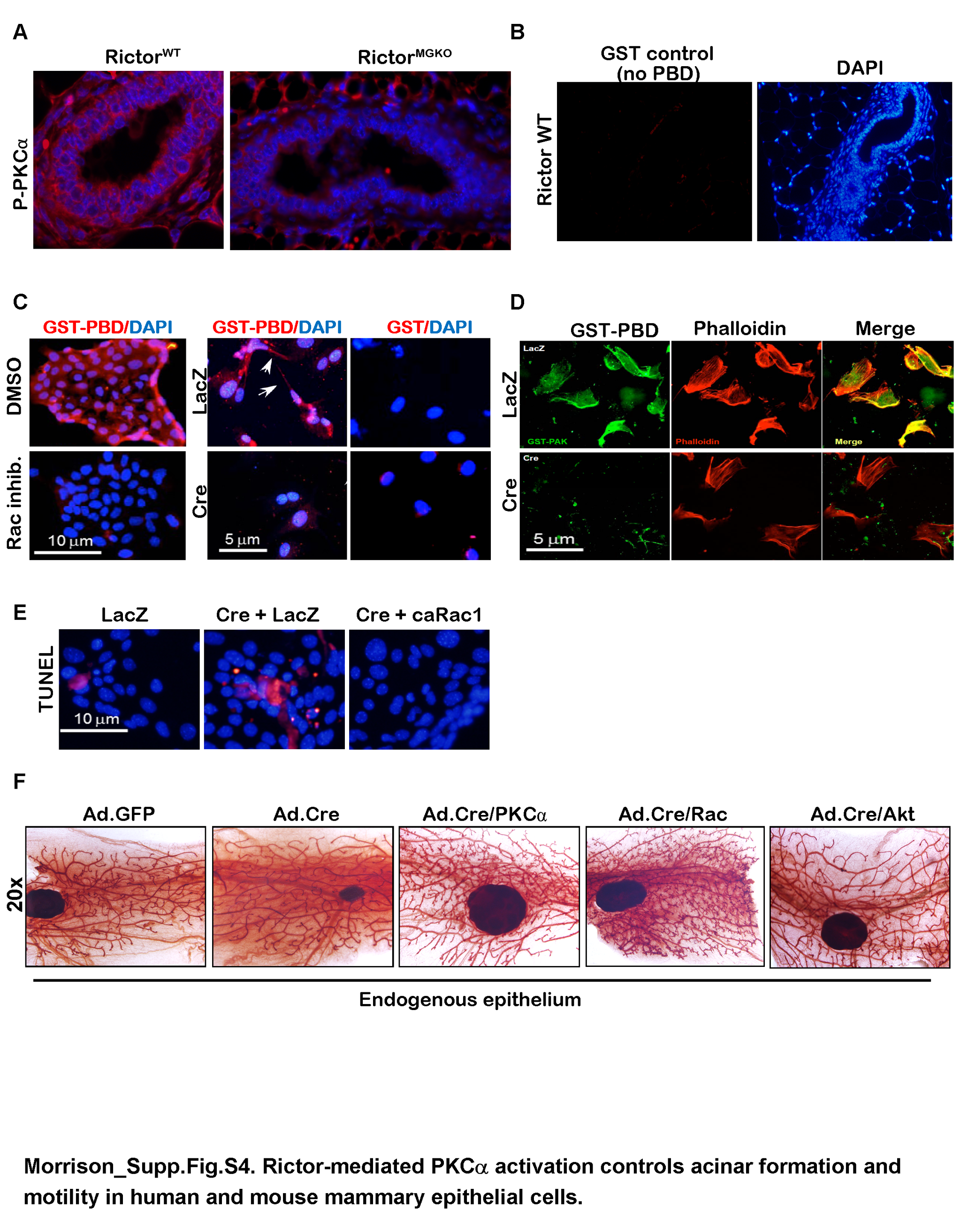

Supplement: S4 Fig — A. Immunofluorescent detection of P-PKC-alpha in mammary gland sections from 6 week old virgin Rictor WT and Rictor MGKO mice. B. In situ detection of GTP-only control (no PBD) via IF detection of GST-PBD (red; nuclei stained with DAPI, blue) on mammary gland sections from 6-week old mice. C. WT PMECs cultured ± Rac inhibitor or Rictor FL/FL PMECs infected with Ad.LacZ or Ad.Cre were assessed for GTP-bound Rac via IF detection of GST-PBD (red; nuclei stained with DAPI, blue). D. Rictor FL/FL PMECs infected with Ad.Cre or Ad.LacZ were probed with GST-PBD (green) and counterstained with phalloidin (red). Representative images are shown. E. Rictor FL/FL PMECs infected with Ad.Cre or Ad.LacZ (±Ad.caRac1) were analyzed for TUNEL. F. Whole mount analysis of endogenous mammary glands harvested from WT recipient mice. (TIF) [file pgen.1005291.s004.tif]

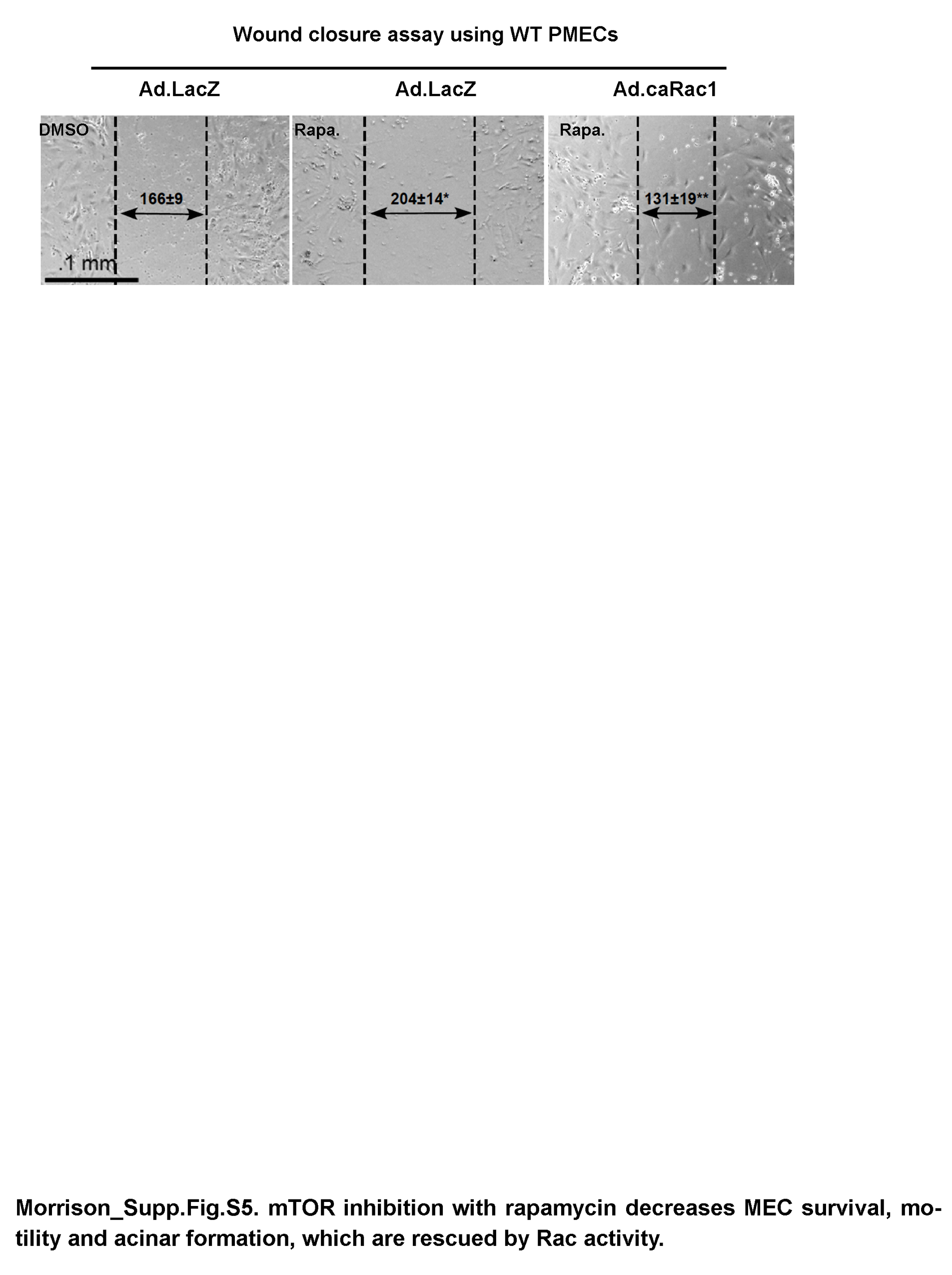

Supplement: S5 Fig — WT PMECs infected with Ad.LacZ or Ad.caRac1 were grown to confluence (3–5 days after infection) then wounded using a P200 pipette tip in the presence of DMSO or rapamycin. Monolayers were imaged after 24 hours and total wounded area (arbitrary units) remaining was measured at 24 hours after wounding. Values shown are the average ± S.D. N = 6 per time point. (TIF) [file pgen.1005291.s005.tif]

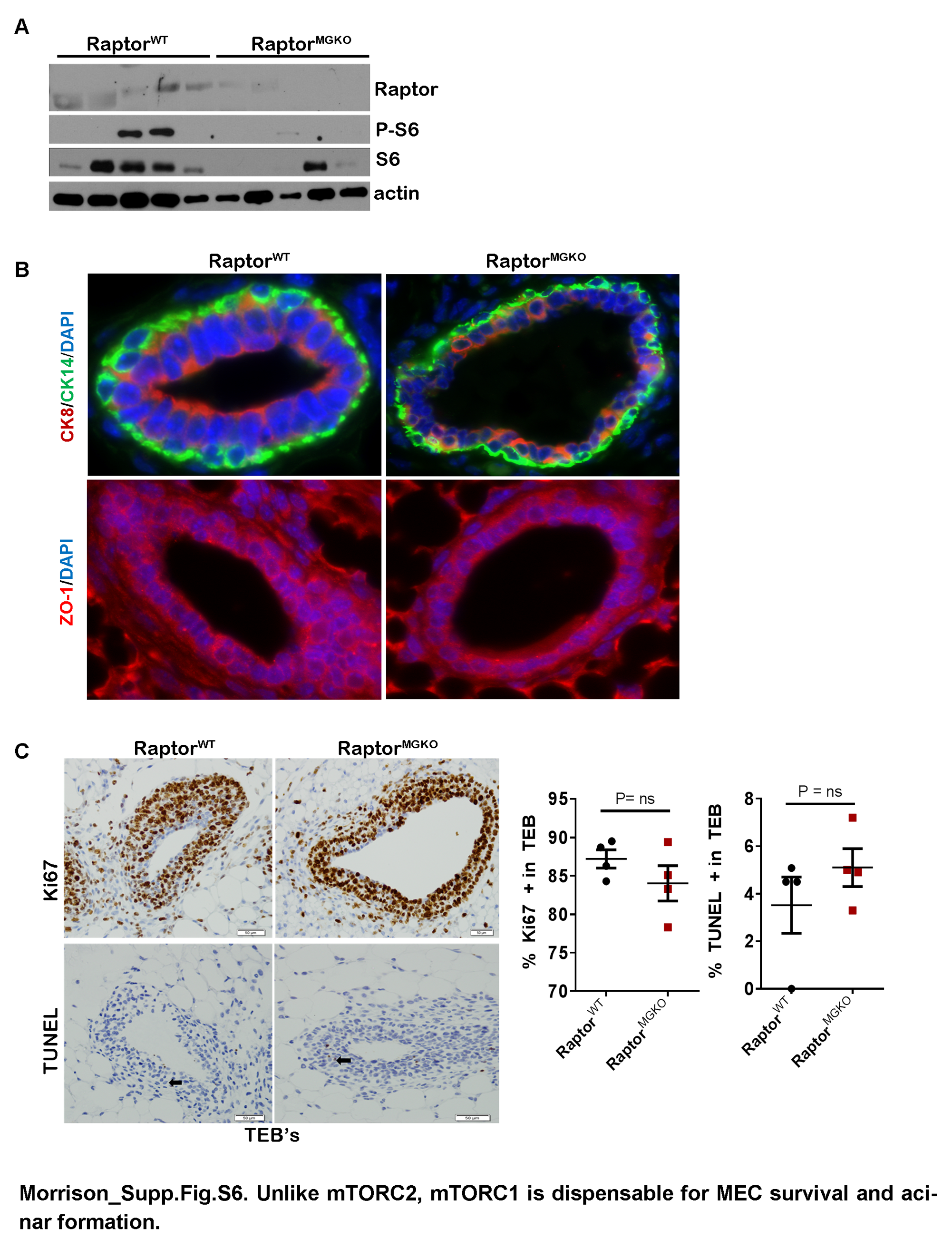

Supplement: S6 Fig — A. Western analysis of whole mammary gland lysates from 10 week old mice. B. IF for CK8, CK14 and ZO-1 in mammary gland sections. C. IHC for Ki67 or TUNEL in TEBs from 6 week old wild-type Raptor WT mice and Raptor MGKO mice. Average percent Ki67+ and TUNEL+ nuclei (± S.D.) was determined, Student’s T-test. (TIF) [file pgen.1005291.s006.tif]
